# Comparative analysis of CDR3 length-dependent patterns in VHHs

**DOI:** 10.3389/fimmu.2025.1647230

**Published:** 2025-08-15

**Authors:** Lu Zhang, Tianyu Yang, Yao Zhang, Jiahui Yan, Jiaguo Li, Wenfeng Xu, Weimin Zhu, Xinhao Wang

**Affiliations:** ^1^ Drug Discovery and Development, Shanghai Cell Therapy Group Co. Ltd., Shanghai, China; ^2^ Drug Discovery and Development, Chantibody Therapeutics, Menlo Park, CA, United States

**Keywords:** CDR3, CDR3 length, VHH, VHH-Ag interaction, epitope, paratope, nanobody, single-domain antibody

## Abstract

**Introduction:**

VHHs, or nanobodies, are distinguished by their compact size, high stability, and unique ability to selectively target specific epitopes. The CDR3 region in VHHs, which plays a crucial role in antigen binding, exhibits significant diversity and varies among species.

**Method:**

This study systematically examined CDR3 length dependent patterns by analyzing NGS sequences from the PBMCs of Alpacas, Llamas and Bactrians, in conjunction with VHH structure data from the public database.

**Results:**

VHHs from Alpacas and Llamas exhibited similar CDR3 length distributions, while Bactrian VHHs displayed significantly longer but narrower length distribution. Key sequence, structural, and VHH/antigen interaction characteristics correlated with CDR3 length were identified. Specifically, longer CDR3s were associated with a lower net charge, reduced surface hydrophobicity, and enhanced interactions with other VHH regions. Structural analyses revealed that longer CDR3s tended to adopt bent conformations with increased helical and coil structures, whereas shorter CDR3s favored extended conformations and β-sheets. Associations between CDR3 length and amino acid usage patterns within VHH sequences were also observed, including preferences at various sites and in antigen interactions. Notably, species-specific differences were apparent, with Alpaca and Llama VHHs showing more pronounced CDR3 length-dependent patterns than those from Bactrians.

**Discussion:**

These findings highlight the significant impact of CDR3 length on VHH sequence, structure, and antigen interaction characteristics, providing valuable insights for VHH engineering, synthetic library design, and the development of therapeutic nanobodies optimized for targeting diverse epitopes.

## 1 Introduction

Heavy-chain-only antibodies (HCAbs), which lack the light chains typically found in conventional antibodies, are naturally present in the immune repertoire of camelids and cartilaginous fish (1). The variable domains of HCAbs, known as VHHs or nanobodies, are fully capable of antigen binding. VHHs have dimensions in the nanometer range and a molecular weight of approximately 15 kDa. They have been extensively studied because of their many applications as research reagents, diagnostic tools, and therapeutic products (2–5). They possess several unique properties compared to conventional antibodies, including a small size, high affinity and specificity, better solubility and thermostability, and the ability to target special epitopes such as cavities. Their single-chain structure also makes them particularly well-suited for developing multi-specific targeting therapies (6, 7).

The complementarity-determining region 3 (CDR3), one of the hypervariable loops in VHHs and heavy chains of conventional antibodies (VHs), is formed through VDJ recombination during B cell development (8, 9). Through genetic recombination, somatic hypermutation, and other diversification mechanisms, CDR3 becomes the most diverse region of antibody sequences in terms of sequence length and amino acid composition, and as a result, plays a critical role in antigen binding (10).

The length of CDR3 varies among antibodies from different species (11), with the longest CDR3 found in Bos taurus antibodies (12). The distribution of CDR3 lengths within a repertoire follows a normal distribution (13, 14). The natural repertoire exhibits certain constraints on the length and composition of CDR3 sequences (15). Some diseases, especially autoimmune diseases, affect the average CDR3 length and composition in the repertoire. The CDR3 is shorter and has higher percentage of charged amino acids in patients with systemic lupus erythematosus than healthy controls (16). Conversely, in rheumatoid arthritis patients, the length of CDR3 is longer (17).

The CDR3 is the primary component of paratope, and different lengths of CDR3 may lead to different structure conformation. As a result, antibodies with different CDR3 lengths are expected to target distinct types of epitopes. For example, many potent broadly neutralizing antibodies against HIV-1 feature long heavy chain CDR3 length (18). Antibodies targeting MUC-1 VNTR repeat epitopes tend to have a preferred CDR3 length (19), while antibodies against small molecules are more likely to have short CDR3 (20).

VHHs are known to have longer CDR3 regions than conventional antibodies (21, 22). However, our recent study (23) demonstrated that some subgroups of VHHs in Alpaca have shorter CDR3 lengths than conventional antibodies. Due to the single-chain nature of VHHs, the CDR3 region may play a more significant role in VHH structure and function, and the length of CDR3 may have a greater impact on VHH sequence, structure features, and VHH/antigen interaction characteristics. In this study, we conducted a comprehensive analysis on CDR3 length-dependent patterns using NGS sequences from the PBMCs of Alpacas, Llamas and Bactrians, along with VHH structural data from the public database. We found that many sequence and structure features were significantly correlated with CDR3 length. VHHs from Alpacas and Llamas showed remarkably similar correlations between sequence/structure features and CDR3 length, whereas VHHs from Bactrians displayed quite different correlation patterns. These findings provide a better understanding of the relationship between CDR3 length and VHH sequence/structure features in the immune repertoire, and will be valuable for VHH engineering, synthetic library design, and VHH-based therapeutics development.

## 2 Materials and methods

### 2.1 VHH sequencing and analysis

PBMCs were collected from 22 Alpacas, 10 Llamas, and 6 Bactrians (**Supplementary Table S1**) prior to immunization. NGS libraries were prepared using primers targeting the VHH hinge region and leader sequences (**Supplementary Table S2**). Libraries were sequenced using MiSeq (Illumina, Inc) with 2x300 PE module. Sequences were processed using ANARCI (24) to identify CDR1/CDR2/CDR3 and framework regions based on IMGT numbering (25). The biophysical and chemical properties of each VHH sequence were subsequently analyzed. To determine the germline origins for each VHH sequence, we aligned alpaca and llama VHH sequences with alpaca germlines downloaded from IMGT (26), and Bactrian VHH sequences with Bactrian germlines determined by Liu et al. (2022) (27). All alignments were conducted using blastn (28) with parameters similar to those of Igblast (29). The net charges of different VHH regions at pH 7.4 were calculated by summing the charges of D (−1), E (−1), R (+1), K (+1), and H (+0.1). Hydropathy indices for each VHH region were calculated by averaging the hydropathy index (30) of each residue within the region. To minimize errors introduced during PCR and sequencing steps, only sequences with at least 5 counts were included. Duplicated sequences were removed to ensure each sequence in the set was unique at the amino acid level. Additionally, to eliminate outliers, only sequences with an absolute CDR3 length z-score of less than 2.5 were retained. A total of 465,173 VHH sequences from Alpaca, 219,381 from Llamas, and 187,873 from Bactrians were included in the analysis.

For the comparative analysis, a set of human VH sequences generated by Ellebedy et al. (2016) (31) was obtained from the Observed Antibody Space (OAS) database (32). Using the same deduplication and outlier filtering procedures applied in the VHH sequence analysis, a total of 325,005 VH sequences were included for the comparative analysis of CDR3 lengths.

### 2.2 Structure dataset and analysis

Crystal structures of antigen-VHH complexes were extracted from SAbDab-nano (33) on September 14, 2024. The data were further processed as follows: First, only complexes with protein antigens and VHH species labeled as originating from alpacas (vicugna pacos), llamas, or camels were retained. Second, complexes with epitopes containing fewer than 6 amino acids were removed to prevent potential false interactions. Third, redundancy was removed based on VHH sequence identity, resulting in a non-redundant structural dataset comprising 633 antigen-VHH complexes: 213 from Alpacas, 328 from Llamas, and 92 from Camels.

CDRs were defined according to the IMGT numbering scheme. Epitope and paratope residues were identified as those with any atom within 4 Å between the antigen and antibody. To calculate the minimum distance between position 42 and CDR3 residues (denoted as FR2_CDR3_Dis) for CDR3 conformation analysis, the central coordinates of the residue at position 42 and each residue in CDR3 (excluding the first and last two residues) were obtained. The distance between two central coordinates was calculated as the distance between two residues.

All structure-based features for VHHs were computed based on 3D structures of the Fv domain. Secondary structure analysis was conducted using the DSSP tool (34), which identifies hydrogen bonds and geometrical arrangements in VHH structures to classify regions into types such asα-helix (H), β-strand (E), 310 helix (G), π-helix (I), turn (T), bend (S), and coil (C). Features related to surface charge were calculated using PEP-Patch (35), which computes the surface charge as the integral of the electrostatic potential over the VHH surface. LPP_area and LNP_area represent the area of largest positive and negative surface patches identified by PEP-Patch, respectively. Surface hydrophobicity was calculated as the sum of the solvent-accessible surface areas (SASAs) of amino acids in the VHH, determined using freeSASA (36), each multiplied by its respective hydrophobicity index (30). The solubility property of VHH was assessed using the total score calculated by AggreScan3D (37), with more negative values indicating better solubility. Therefore, when calculating the correlation with CDR3 length, the negative value of the score was used.

Interaction interface features were determined using 3D structures of antigen-VHH complexes. To calculate the buried surface area at the interface, freeSASA (36) was used to compute the SASA for both the antigen and VHH in their complex and monomeric forms. The buried surface area of interface was then derived by subtracting the SASA of the complex from the sum of the SASA of the antigen and VHH in their monomeric forms. Similarly, for the antigen (epitope) and VHH (paratope), the buried surface area was calculated by subtracting the SASA in the complex from that in the monomeric forms. Arpeggio (38) was used to determine interaction types for each complex using a distance threshold of 4 Å. Interatomic interactions between VHH and antigen were considered, with each type of interaction counted separately if an atom pair established more than one type of interaction.

### 2.3 Statistical tests

To assess significant correlations between groups, we calculated Pearson’s correlation coefficient r and performed paired correlation test. To compare two groups of data, we mainly used the two-tailed Mann-Whitney U test to assess significant differences, except those mentioned in the text. Benjamini-Hochberg (BH) procedure was used to control the False Discovery Rate (FDR) in multiple testing corrections. A P-value of less than 0.05 is considered significant. In figures, P-values are shown as follows: ns: P > 0.05; *0.01< P <=0.05; **0.001 <= P < 0.01; ***0.0001<= P < 0.001; ****P <= 0.0001.

### 2.4 Uniform Manifold Approximation and Projection analysis

The AntiBERTy language model (39) was used to generate embeddings for the sequences using “embed” method as recommended by the author. Per residue embeddings were further averaged along the length of input sequence, and resulted vectors were used as input for UMAP analysis.

## 3 Results

### 3.1 Comparisons of CDR length and its distribution

**Figure 1:**
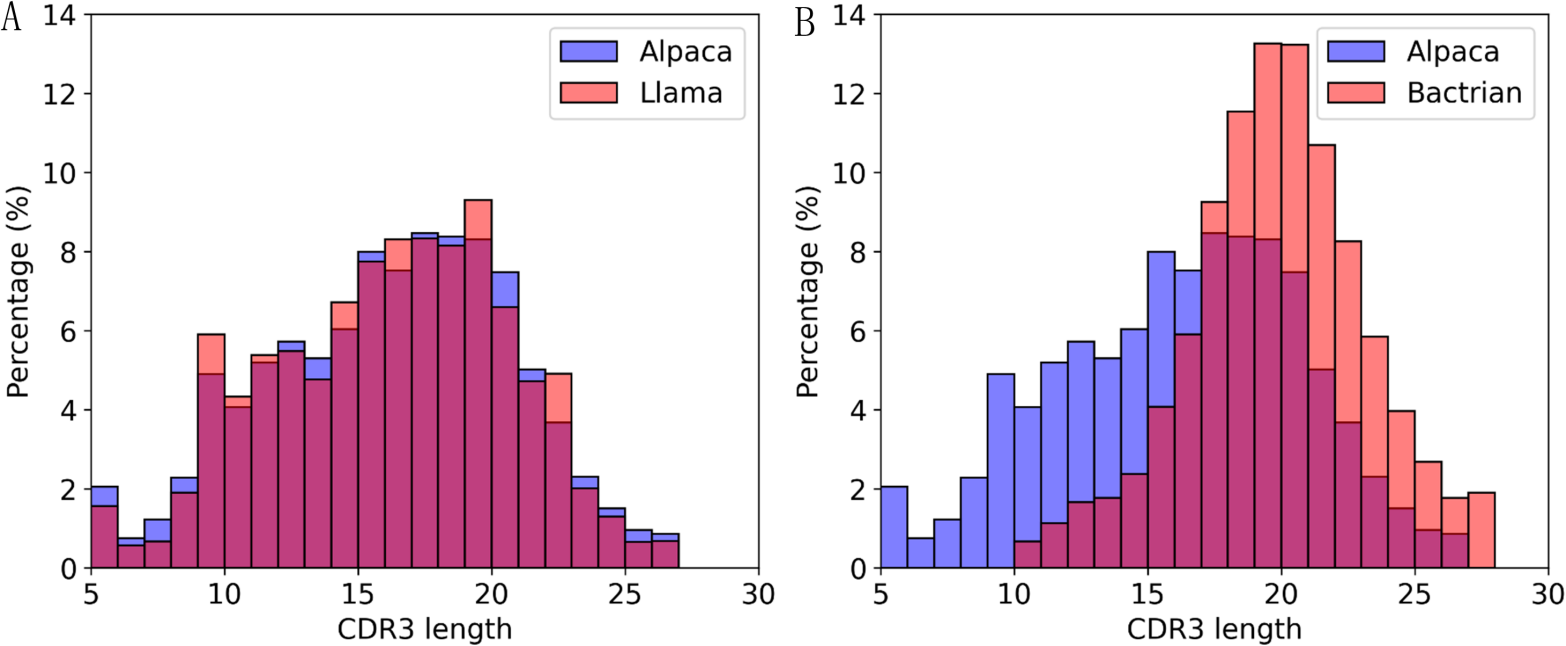
CDR3 length distribution. Alpaca and Llama exhibited similar CDR3 length distributions **(A)**. Alpaca had a wider CDR3 length distribution but, on average, a shorter CDR3 length compared to Bactrian **(B)**.

Using NGS data from PBMCs of three species, we compared the average length of CDR3 and its distribution. VHHs from Alpaca and Llama exhibit highly similar average CDR3 length, while VHHs from Bactrian have significantly longer CDR3 (**Table 1**, P < 0.0001), with an average length 3.6 amino acid longer. VHHs from Alpaca and Llama also displayed similar CDR3 length distribution with standard deviations of 4.61 and 4.47 respectively (**Table 1**, **Figure 1A**). Interestingly, VHHs from Bactrian have significantly narrower CDR3 length distribution, with a standard deviation of 3.36 (F=1.88, P < 0.0001, **Figure 1B**). These results suggest that VHHs from different camelid species have distinct CDR3 length and distribution. Previous studies (1) have shown that more than 50% of antibodies in Bactrian repertoire are VHHs, while VHHs in Alpaca and Llama make up only 20-30% of their repertoire. This may indicate that the wider variation of CDR3 length in Alpaca/Llama VHHs contributes to higher diversity, potentially compensating for their lower abundance in repertoire. For comparison, we also analyzed the same properties for CDR3 from Human VH sequences. As expected, the CDR3 length in Human VH sequences is significantly shorter than that in VHHs (P < 0.0001), being 1.6 residues shorter compared to CDR3 length in VHHs from Alpaca and Llama, and 5.2 residue shorter compared to CDR3 length in VHHs from Bactrian (**Table 1**). Additionally, the CDR3 length distribution in Human VH sequences exhibits narrower distribution, with a standard deviation of 3.81, compared to Alpaca and Llama VHHs (F=1.46, P < 0.0001, **Supplementary Figure S1A**), but wider distribution than that in Bactrian VHHs (F=0.78, **Supplementary Figure S1B**).

**Table 1:** Distinct CDR3 length in VHHs and human VH.

Species	Alpaca	Llama	Bactrian	Human
Count	465173	219381	187873	324005
Mean ± SE	15.73 ± 0.01	15.74 ± 0.01	19.34 ± 0.01	14.11 ± 0.01
Median	16	16	19	14
STD	4.61	4.47	3.36	3.81

Mean ± SE represents the mean ± standard error, and STD refers to the standard deviation.

Because of its single-chain nature, the CDR3 may play a more significant role in shaping the sequence and structure characteristics of VHHs. Given the wide variation of CDR3 length, especially in VHHs from Alpaca and Llama, significant differences in sequence, structure, and VHH-antigen interactions are expected between VHHs with short and long CDR3 lengths. To explore this possibility, we systematically analyzed the correlations between sequence, structure features and CDR3 length throughout the rest of this study.

### 3.2 CDR3 length-dependent sequence charges and hydropathies

Biophysical properties of VHHs or antibodies, such as charge and hydropathy, are known to play a key role in their solubility, stability, and antigen interaction, ultimately influencing their therapeutic efficacy and safety.

**Figure 2:**
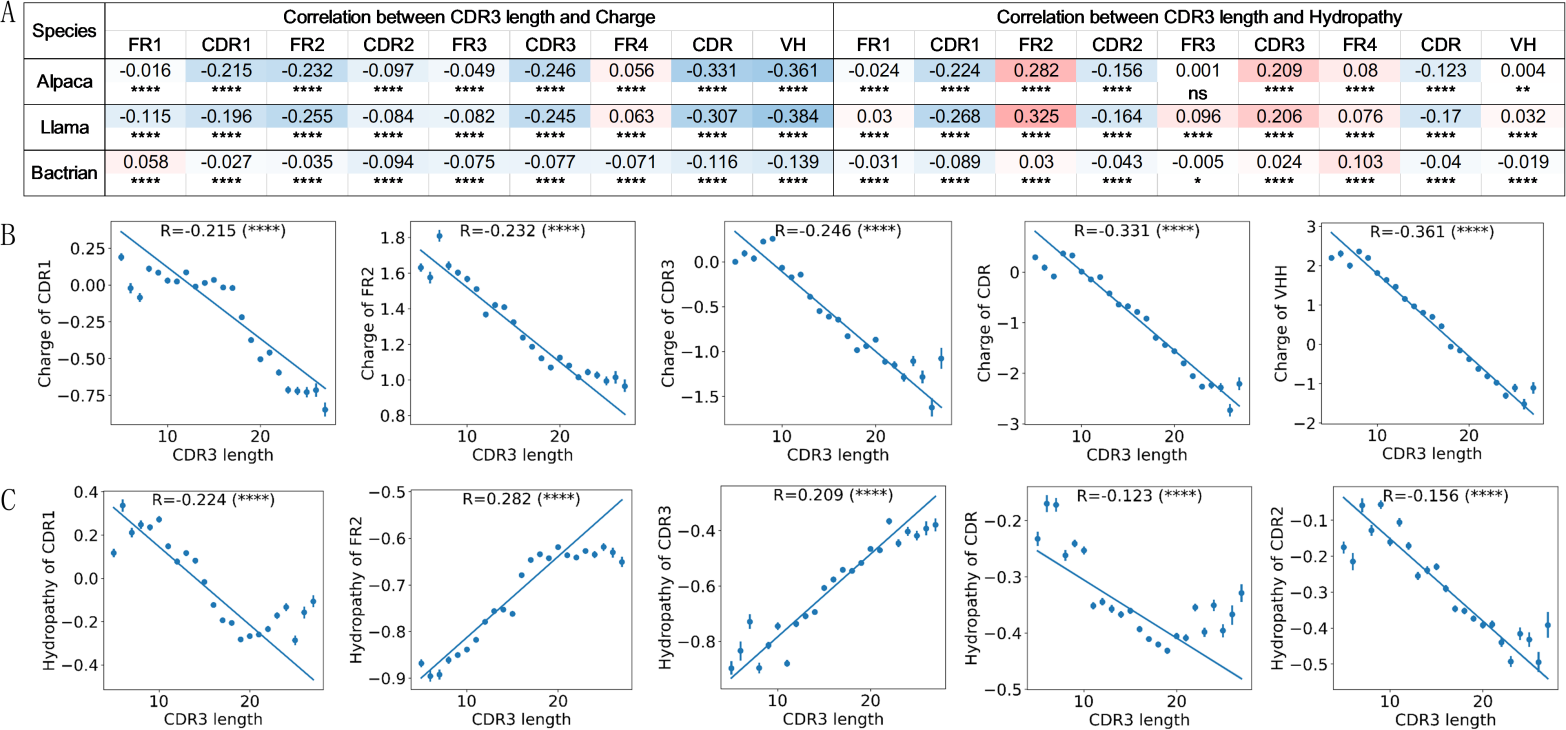
Significant correlations between CDR3 length and charge or hydropathy. Pearson correlations and significance indicators for the charge and hydropathy in regions of VHHs in relation to CDR3 length across species **(A)**. CDR3 length is negatively correlated with the charge in CDR1, FR2, CDR3, CDRs, and full VHH **(B)**. CDR3 length is negatively correlated with the hydropathy of CDR1 and CDR2, CDRs, positively correlated with FR2 and CDR3 **(C)**. Y-axis represents the mean values of features at each CDR3 length, with error bars indicating variability. Pearson correlation coefficients were calculated based on all data points, and regression line based on mean values. P-values are marked as followings: ns: P > 0.05; *0.01< P <=0.05; **0.001 <= P < 0.01; ****P <= 0.0001.

Significant negative correlations were observed between CDR3 length and charge in all regions, except FR4 for VHHs from Alpaca and Llama, and FR1 for VHHs from Bactrian (**Figure 2A**). The charge in CDR1, FR2, CDR3, CDR, and VHH displayed more pronounced negative correlation with CDR3 length (**Figures 2A, B**). VHHs from Alpaca and Llama display similar negative correlation patterns (**Figure 2A**). In contrast, Bactrian VHHs show a weaker correlation, with values below 0.1 across many regions.

Hydrophobic indices also showed some significant CDR3 length-dependent patterns (**Figures 2A, C**). Similar to charge, the hydropathy correlation patterns in Alpaca and Llama VHHs are similar to each other and have higher correlations than those in Bactrian camels. For VHHs from Alpaca and Llama, a significant negative correlation exists between CDR3 length and the hydropathy of CDR1 and CDR2, indicating longer CDR3s are associated with more hydrophilic CDR1 and CDR2 regions. In contrast, a positive correlation is observed between hydropathy of FR2 and CDR3, where longer CDR3s correspond to more hydrophobic regions (**Figure 2C**). Bactrian camels follow a similar trend but with lower correlation coefficients, likely due to their narrower CDR3 length distribution (**Figure 1B**).

Our previous study (23) demonstrated that VHHs in Alpaca can be classified into several types, largely based on germlines VHHs used, with significantly different CDR3 length and biophysical properties. By analyzing VHHs from both Alpaca and Llama, we observed that different V gene germlines exhibit preferences for specific CDR3 lengths in VHHs (**Supplementary Figure S2A**). However, such preference was not significantly evident in VHHs from Bactrian. These results help explain some of the observed correlation patterns. For instance, Alpaca VHHs derived from IGHV3S65*01 have the longest CDR3 regions, compared to IGHV3S53*01-derived VHHs, which have the shortest. IGHV3S65*01 contains negatively charged residues: D (IMGT35) in CDR1 and E (IMGT49) in FR2. Similarly, IGHV3S61*01 and IGHV3S66*01, which are associated with longer CDR3s, also contain these residues, along with an additional D (IMGT62) in CDR2. This resulted in a higher negative charge in VHHs derived from these germlines (**Supplementary Figure S2**), helping to explain the observed negative correlation between CDR3 length and charge. However, the negative correlation between charge and length observed in the CDR3 region cannot be solely attributed to the germline these VHHs are derived from, as CDR3 region is the result of VDJ recombination. Some selective pressures during antibody repertoire evolution may be responsible for this pattern.

**Figure 3:**
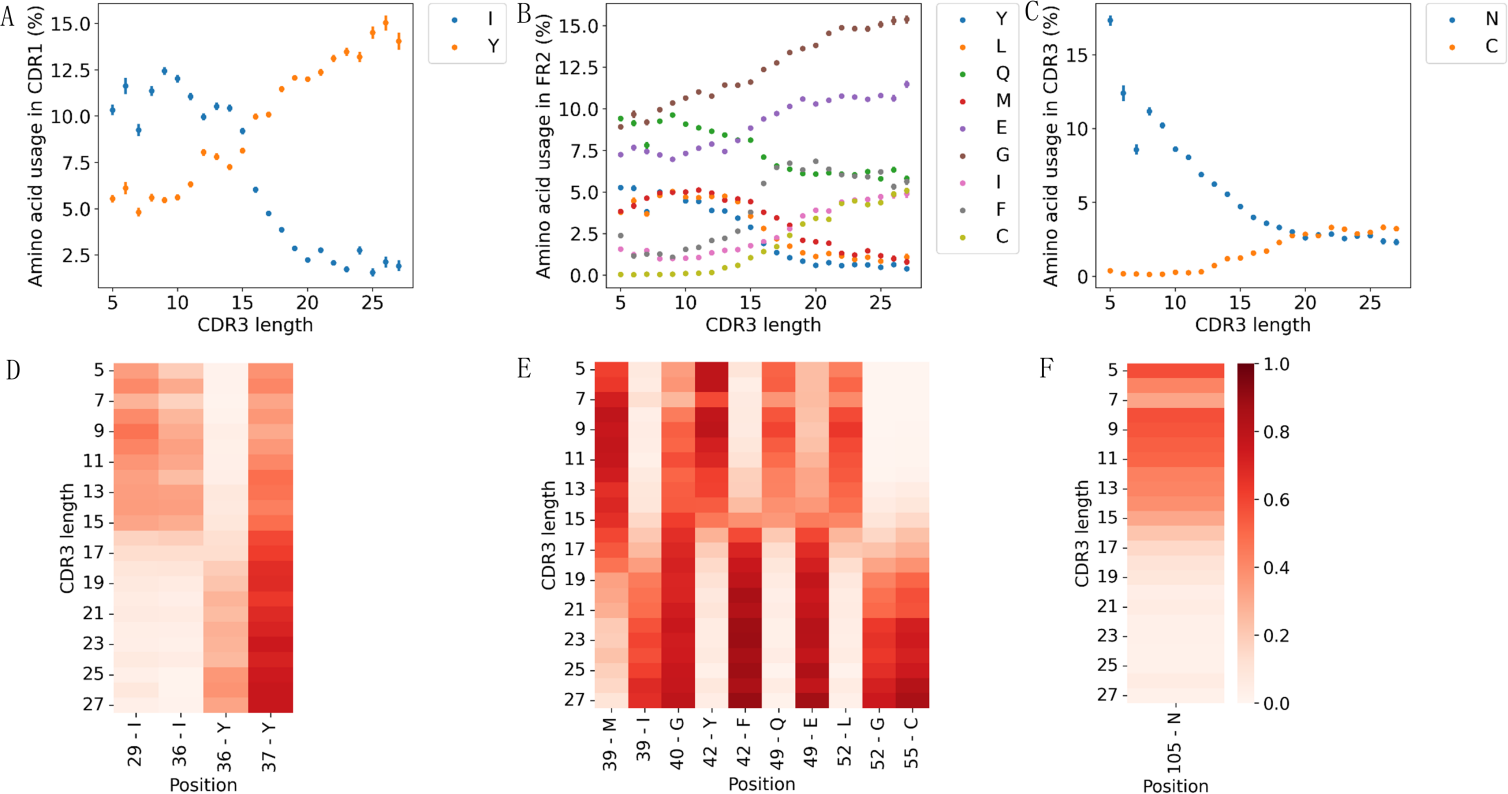
Amino acid usages associated with CDR3 length in Alpacas. VHHs with longer CDR3s exhibited increased usage of Y and decreased usage of I in the CDR1 region **(A)**. CDR3 length negatively correlated with Y, L, Q, M, and positively correlated with E, G, I, F, C in the FR2 **(B)**. VHHs with longer CDR3s showed higher usage of C and lower usage of N in the CDR3 region **(C)**. All correlations were significant (P-value < 0.0001). Heatmaps displayed significant correlations between amino acid residue usages at specific positions with CDR3 length for CDR1 **(D)**, FR2 **(E)**, and CDR3 **(F)**.

Similarly, some correlation patterns between CDR3 length and hydropathy can be explained by the germlines used by VHHs. For instance, IGHV3S53*01, which is used mostly by VHHs with short CDR3, has the highest hydropathy value in CDR1 and CDR2, and the lowest in FR2 (**Supplementary Figure S2B**). This explains why VHHs with short CDR3 tend to have more hydrophobic CDR1 and CDR2 regions and more hydrophilic FR2. The strong correlation between CDR3 hydropathy and CDR3 length cannot be explained by the germline these VHHs used, as the CDR3 region results from VDJ recombination. However, the likely presence of the hydrophobic residue C with a hydropathy index of 2.5, in longer CDR3 region as shown in next result section (**Figure 3C**), may account for some of the observed positive correlation.

### 3.3 CDR3 length-dependent amino acid usages

To further investigate CDR3 length-dependent sequence features, we analyzed changes in amino acid usage across different segments of VHHs as CDR3 length varies. As observed previously, the amino acid usage patterns in Alpaca and Llama VHHs are highly similar, particularly in the CDR1, FR2 and CDR3 regions (**Figure 3**, **Supplementary Figure S3**, **Supplementary Tables S3**, **S4**). In VHHs from Alpacas (**Figure 3**, **Supplementary Table S3**), the frequency of residue Y in CDR1 increases with longer CDR3, while the frequency of residue I decreases (**Figure 3A**). This trend is more evident at positions 29, 36, and 37 (IMGT numbering) (**Figure 3D**). In the FR2 region, the usage of more amino acids is found to correlate with CDR3 length (**Figures 3B, E**). Specifically, the usage of Y, L, Q, and M showed a significant negative correlation with CDR3 length, while the usage of G, I, F, E, and C showed positive correlations. There are four pairs of residues at the same IMGT positions, 39, 42, 49 and 52, showing opposite correlations with CDR3 length (**Figure 3E**). For example, at IMGT position 42, VHHs with shorter CDR3 predominantly use Y, while VHHs with longer ones use F, which is consistent with our previous findings (23). In the CDR3 region, VHHs with longer CDR3s have a higher proportion of amino acid C but less N, with the length dependency of amino acid N being most evident at position 105 (**Figures 3C, F**), while no site-specificity is observed for C.

These patterns can be partially attributed to the germlines these VHHs used. For example, IGHV3S65*01, which is more likely found in VHHs with long CDR3 (**Supplementary Figure S2**), contains Y at positions 36 and 37, I at position 39, F at position 42, E at position 49, G at position 52, and C at position 55, consistent with the observed positive correlations between the usage of these residues and CDR3 length. Conversely, IGHV3S53*01, typically associated with VHHs with short CDR3, contains amino acids that are negatively correlated with CDR3 length at the aforementioned positions and includes residue N at position 105 of the CDR3.

Furthermore, these amino acid usage patterns align with the previously observed hydropathy trends. For instance, VHHs with short CDR3 prefer hydrophobic amino acid I in CDR1, while VHHs with longer CDR3 prefer hydrophilic amino acid Y. This results in a negative correlation between CDR1 hydrophobicity and CDR3 length.

Similar correlations between CDR3 length and amino acid usage are observed in VHHs from Llamas (**Supplementary Figure S3**, **Supplementary Table S4**). In contrast, VHHs from Bactrian display weaker and different correlations between CDR3 length and amino acid usage, with some similarities to VHHs from Alpaca and Llama at certain positions in FR2, such as positions 42 and 49 (**Supplementary Figure S4**, **Supplementary Table S5**).

### 3.4 CDR3 length-dependent VHH structural characteristics

**Figure 4:**
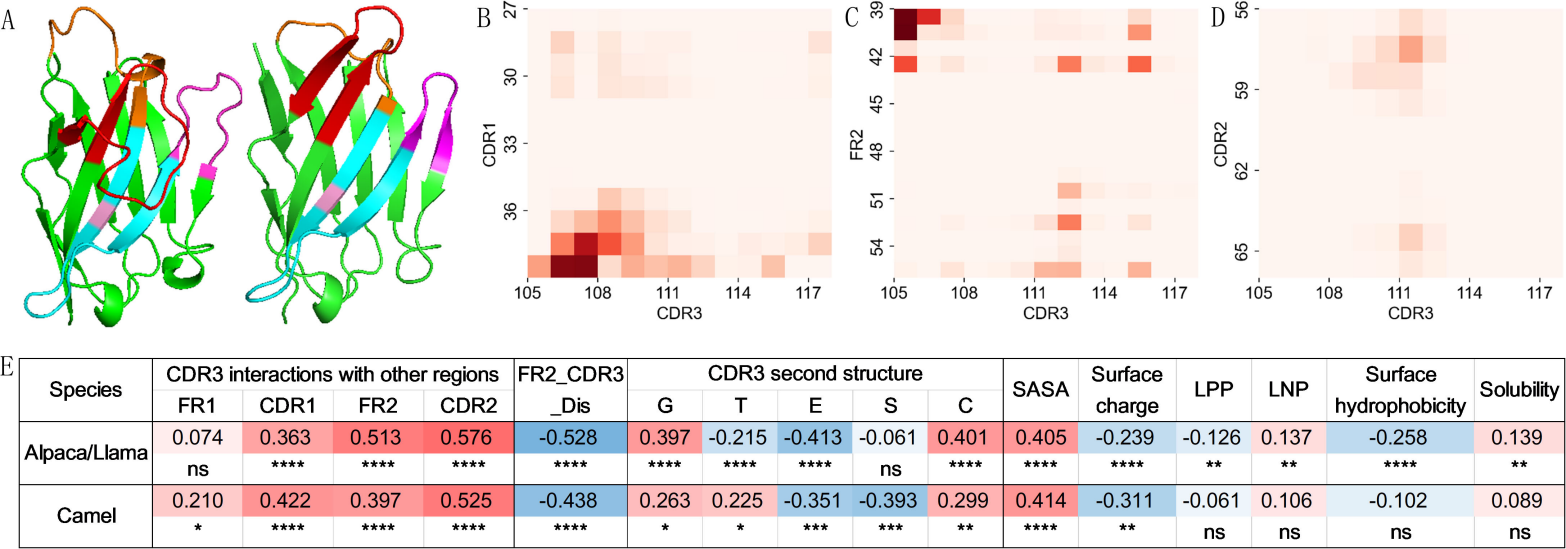
CDR3 length-dependent structural patterns in VHH. **(A)** Examples of VHHs with bent down (longer CDR3, PDB ID: 5U4L) and extended (shorter CDR3, PDB ID: 6U55) CDR3 conformation. The CDR3 region is colored red, with CDR1, CDR2, and FR2 colored in orange, magenta, and cyan, respectively. The residue at IMGT 42 is highlighted in pink. Heatmaps show interaction probabilities between CDR3 and CDR1 **(B)**, FR2 **(C)**, and CDR2 **(D)**. Pearson correlation coefficients and significance indicators for structural features of VHHs in relation to CDR3 length across species **(E)**. FR2_CDR3_Dis: distance between CDR3 and the FR2 region, calculated as the minimum distance between the residue at IMGT 42 and any residue within CDR3 after excluding first and last two residues. LPP, largest positive patch; LNP, largest negative patch.

Previous studies have shown that CDR3s with different lengths may adopt distinct conformations (23, 40, 41). Specifically, longer CDR3 regions tend to adopt a bend down conformation, whereas shorter CDR3 regions are more likely to assume an extended conformation (**Figure 4A**). To further investigate the relationship between CDR3 length and VHH structural characteristics, VHH structural data were collected from SAbDab-nano (33) and analyzed. The full list of the structural dataset is available in **Supplementary Table S6**. Due to the limited amount of structural data and the high similarity between VHHs from Alpaca and Llama, based on our current sequence analysis results and previous study (23), we combined the data from Alpaca and Llama into a single group for comparison with Camel data in the structural feature analysis.

Firstly, we investigated the residue interactions between CDR3 and other segments of VHH. Using a distance threshold of 4 Å, we found that CDR3 interacts extensively not only with itself but also with the other two CDRs and FR1 and FR2 regions in both datasets (**Supplementary Figure S5**, **Figures 4B–D**). Major interactions were observed between the starting residues of CDR3 and CDR1/FR2 connecting region, which may not be surprising given their structural proximity. C β-strand connecting to the end of CDR1, is next to F β-strand which connects to the starting of CDR3 based on typical V-domain structures (42). Interestingly, the first few residues of FR1 interact with starting and end residues of CDR3, suggesting that they may be part of paratopes. Indeed, these same FR1 residues have been shown to be involved in antigen binding (43). Further analysis demonstrated a significant positive correlation between the number of interactions and CDR3 length for CDR1, FR2 and CDR2 in Alpaca/Llama, and FR1, CDR1, FR2, CDR2 in Camel, with the strongest correlation observed in the CDR2 region (R > 0.5, P < 0.0001). This might be due to shorter CDR3 being more likely to extend away from the FR2 and CDR2 regions, while longer CDR3 tends to bend toward these regions (**Figure 4A**). Indeed, we observed that the minimum distance between longer CDR3 and the FR2 region (FR2_CDR3_Dis) was significantly shorter (R = -0.528 for Alpaca/Llama and -0.438 for Camel, P < 0.0001), as demonstrated by calculating the minimum distance between the residue at IMGT 42 and any residue within CDR3 after excluding the first and last two residues (**Figure 4E**). The positive correlation between CDR3 length and its interactions with others may not be surprising, as longer CDR3 provide more residues for interaction.

To better understand the link between CDR3 length and its conformation, we analyzed the secondary structures of the CDR3 region using the DSSP tool (34). With high similarity across species, the CDR3 region predominantly adopts five types of secondary structures: 3-turn helix (G), turn (T), β-sheet (E), bend (S), and coils (C) (**Supplementary Figure S6A**). However, there is a significant difference of β-sheet usage between two groups (P < 0.01) (**Supplementary Figure S6A**). Our correlation analysis revealed that CDR3 length is positively associated with the frequency of helix and coils and negatively associated with β-sheet. These correlations were more significant in Alpaca/Llama compared to Camel. Notably, in Alpaca/Llama, CDR3 length also showed a negative correlation with the frequency of turn usage, whereas in Camel, it was positively correlated. Additionally, a significant negative correlation was found between CDR3 length and bend usage in Camel (**Figure 4E**). A turn can be considered as a specific type of bend with more sharp curvature, which is stabilized by hydrogen bond interaction. For Camel VHHs, S type (bend) decreases while T type (turn) increases in CDR3 region as CDR3 length increases, suggesting that longer CDR3s are more likely to form sharp turns. Overall, these results suggest that CDR3s of different lengths exhibit distinct preferences for secondary structure, highlighting specific differences between species.

To understand the potential roles of CDR3 length in VHH structure properties, we investigated the relationship between CDR3 length and the overall structural characteristics of the VHHs (**Figure 4E**). In VHHs from Alpaca/Llama, longer CDR3s were associated with a larger solvent-accessible surface area (SASA) of the VHH. Analysis of the surface electrostatic potential revealed a negative correlation between CDR3 length and the overall surface electrostatic potential, as well as a negative correlation with the area of the largest positive patch (LPP) and a positive correlation with the size of the largest negative patch (LNP). These structural charge characteristics are consistent with previous sequence analyses, which showed a negative correlation between CDR3 length and charge (**Figure 2**). When examining surface hydrophobicity, we found that VHHs with longer CDR3 exhibited significantly lower surface hydrophobicity indices. Previous sequence analyses indicated a negative correlation between CDR3 length and the hydrophobicity of CDR1 and CDR2, and a positive correlation with the hydrophobicity of FR2 and CDR3 (**Figure 2**). Interestingly, further analysis revealed that longer CDR3 regions engaged in more hydrophobic interactions with FR2 (**Supplementary Figure S7**), possibly adopting a “bend down” conformation that buries hydrophobic amino acids in these regions. This structural adaptation likely explains the observed negative correlation between CDR3 length and overall surface hydrophobicity of VHHs. We also observed a slightly positive correlation with VHH solubility (**Figure 4E**). For Camel-derived VHHs, similar CDR3 length dependencies were observed for only SASA and surface electrostatic potential features, consistent with those seen in Alpaca/Llama (**Figure 4E**).

**Figure 5:**
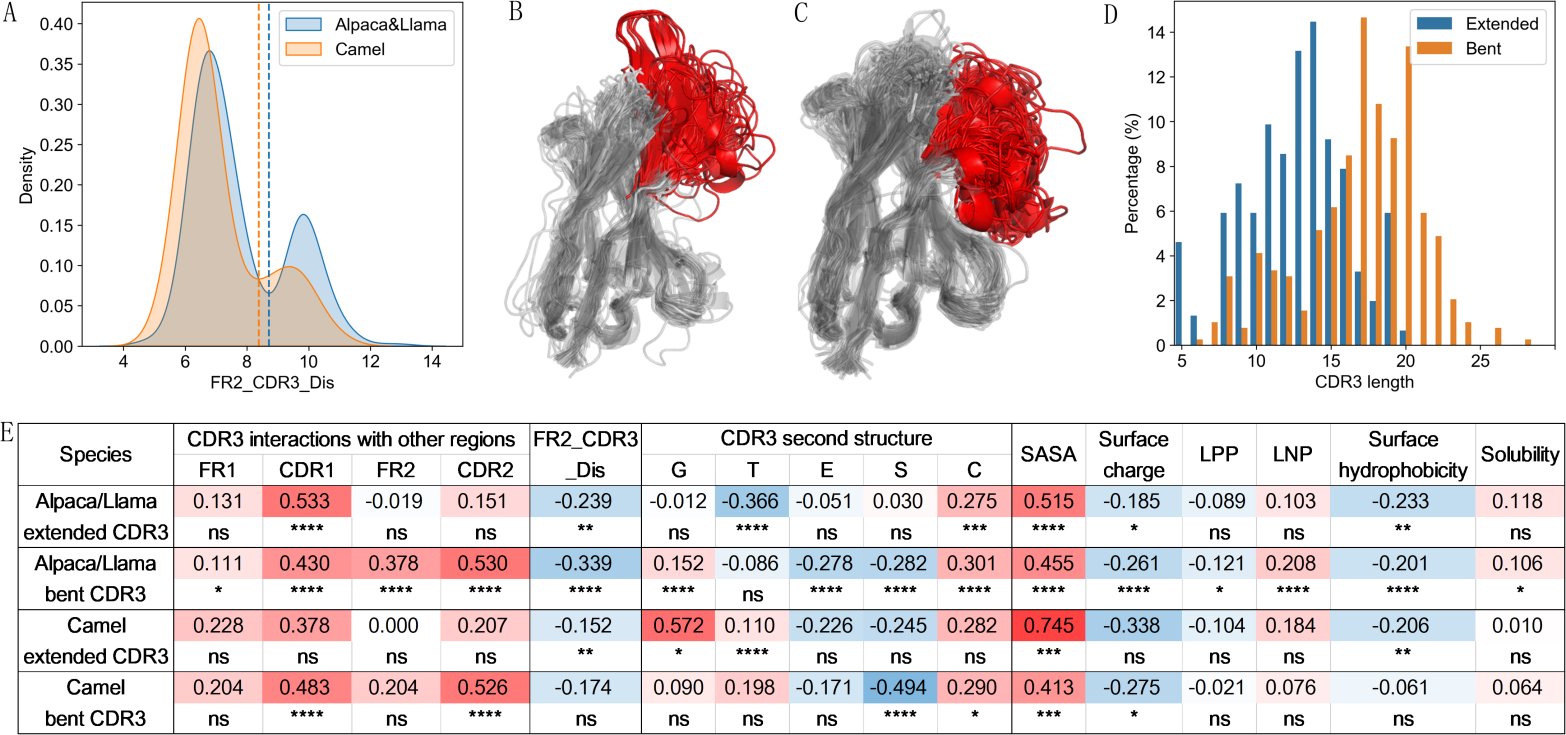
CDR3 length-dependent structural patterns in VHHs with distinct CDR3 conformations. Density plot **(A)** of FR2_CDR3_Dis shows two peaks corresponding to extended CDR3 **(B)** and bent CDR3 **(C)** conformations in Alpaca/Llama VHHs. VHH structures from each peak were superposed, with CDR3 region colored red. VHHs with longer CDR3 are more likely to adopt bent conformation **(D)**. Pearson correlation coefficients and significance indicators for structural features of VHHs in relation to CDR3 length across species **(E)**. FR2_CDR3_Dis: distance between CDR3 and the FR2 region, calculated as the minimum distance between the residue at IMGT 42 and any residue within CDR3 after excluding first and last two residues. LPP, largest positive patch; LNP, largest negative patch.

As mentioned above, VHH CDR3 may exhibit different CDR3 conformations (23, 40, 41). Theoretically, an extended CDR3 maintains a greater distance from FR2, while a bent CDR3 is positioned closer to FR2. To differentiate VHHs with different CDR3 conformations, we used our previously established metric, FR2_CDR3_Dis, which calculates the minimum distance between the amino acid at position 42 in FR2 and any amino acid in CDR3, excluding the first and last two residues. Using this metric, we identified two distinct peaks in the density distribution of VHHs from both datasets (**Figure 5A**). Using the value of FR2_CDR3_Dis at the trough between these peaks as a threshold, VHHs can be categorized into bent (left peak) and extended (right peak) CDR3 conformations. Structural verification confirms the accuracy of this classification metric (**Figures 5B, C**, **Supplementary Figures S8A, B**). Consistent with previous findings (23, 40, 41), our dataset reveals a significant connection between CDR3 length distribution and its conformations, with bent CDR3 more likely to be associated with longer lengths, and extended CDR3 more likely to be associated with short lengths (**Figure 5D**, **Supplementary Figure S8C**).

Within each CDR3 conformation group, we examined VHH structural characteristics and their correlation with CDR3 length. For the interaction characteristics between CDR3 and other segments, VHHs with extended CDR3 exhibited interaction between CDR3 and FR1, FR2, CDR1 and CDR3, with no or low interaction with CDR2. In contrast, VHHs with bent CDR3 showed interaction of CDR3 with all these segments (**Supplementary Figure S9**). Correlation analysis showed that VHHs with extended CDR3 displayed a positive correlation with CDR3 length only for the CDR1/CDR3 interaction, while VHHs with bent CDR3 showed highly significant positive correlation with CDR1, FR2 and CDR2 (**Figure 5E**), similar to the result using the entire dataset (**Figure 4E**). The minimum distance to FR2 (FR2_CDR3_Dis) decreases as CDR3 length increases, particularly for VHHs with bent CDR3 from Alpaca/Llama (**Figure 5E**), consistent with the results using the entire dataset (**Figure 4E**). For the secondary structural features of CDR3 (**Figure 5E**), notable differences in secondary structure usage were observed between CDR3s with different conformations. CDR3s with extended conformation employed more β-sheets (E) and nearly no helices (G), while CDR3s with bent conformation in Alpaca/Llama dataset used more bends (S) and coils (C) (**Supplementary Figures S6B, C**). Correlation analysis with CDR3 length indicated distinct patterns in secondary structure usage. For Alpaca/Llama VHHs with extended CDR3, CDR3 length negatively correlated with the use of turns (T), while VHHs with bent CDR3, the usage of helices (G) positively correlated with CDR3 length, and the usage of β-sheets (E) and bends (S) negatively correlated. In Camel VHHs, CDR3s with extended conformation showed positive correlation between CDR3 length and the usage frequency of helices (G) and turns (T), while CDR3s with bent conformation showed negative correlation for the usage of bends (S). Overall, VHHs with different CDR3 conformations exhibit similar CDR3 length-dependent features, consistent with the patterns observed across the entire dataset (**Figures 5E**, **4E**).

Overall, these findings demonstrate a strong correlation between CDR3 length and its conformations, intra-molecular interaction preferences, and VHH structural characteristics. Moreover, the association patterns for certain features show differences that are both species-specific and, to some extent, conformation specific.

### 3.5 CDR3 length-dependent VHH/antigen interface characteristics

**Figure 6:**
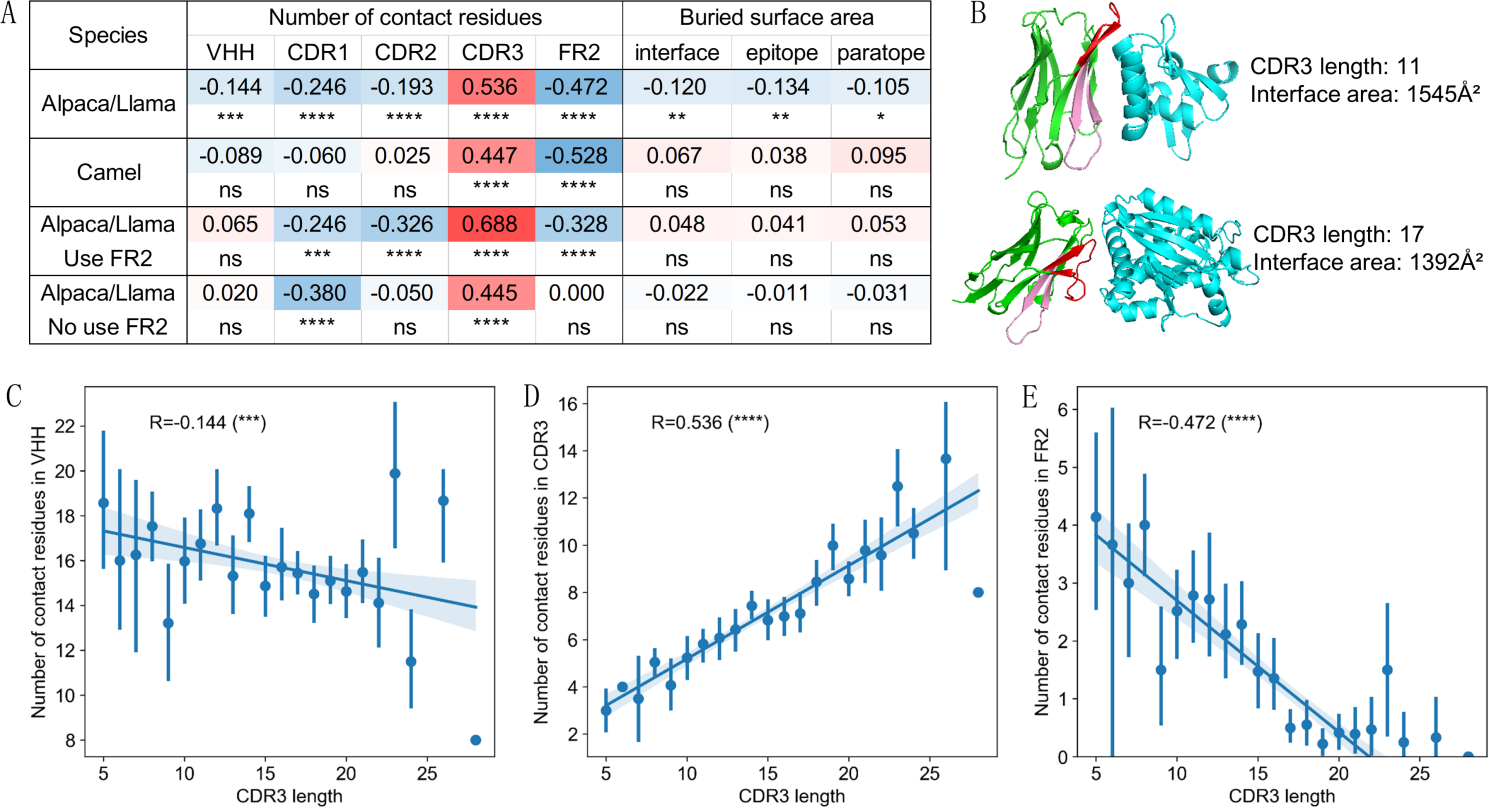
CDR3 length-dependent interaction interface patterns. Pearson correlation coefficients and significance indicators for interface features of VHHs in relation to CDR3 length across species and subtypes **(A)**. Example of VHHs with shorter CDR3s (upper, PDB ID: 6U55) exhibited a relatively larger interaction surface compared to those with longer CDR3s (lower, PDB ID: 5U4L) by involving FR2 residues **(B)**. Scatter plots with linear regression lines illustrating the relationship between CDR3 length and number of contact residues in full VHH **(C)**, CDR3 **(D)**, and FR2 **(E)**. The Y-axis represents the mean values at each CDR3 length, with error bars indicating variability. Pearson correlations were calculated based on all data points, while regression lines were derived from mean values. P-value significance: ns: P > 0.05; *0.01< P <=0.05; **0.001 <= P < 0.01; ****P <= 0.0001.

By analyzing VHH-antigen complexes in our dataset, we investigated the interaction interface characteristics of VHHs with varying CDR3 lengths. In Alpaca/Llama VHHs, a modest inverse correlation was observed between CDR3 length and interaction interface size, quantified by both the number of contact residues and buried surface area (**Figures 6A, C**). A detailed analysis of contact residues revealed distinct correlation patterns across various VHH segments. As expected, the number of contact residues in CDR3 showed a significant positive correlation with its length (R = 0.536, P < 0.0001) (**Figure 6D**). In contrast, other regions, notably CDR1, CDR2, and FR2, exhibited significant negative correlations, especially FR2 (R = -0.472, P < 0.0001) (**Figure 6E**). This can be attributed to the differential interaction modes of VHHs with varying CDR3 lengths: longer CDR3s, while involving more CDR3 amino acids in interactions, are more likely to mask FR2 due to increased interaction with FR2 (**Figure 4E**), thus reducing FR2’s contribution to the interface. Conversely, VHHs with shorter CDR3 expose more FR2 residues, enhancing their involvement in interactions and resulting in relatively larger interaction interfaces (**Figure 6B**). In Camel VHHs, a similar CDR3 length-dependent pattern was observed only in the CDR3 and FR2 regions, despite the high similarity in contact residue distribution between VHHs from these two species (**Supplementary Figure S10**).

Given the unique role of FR2 in VHH-antigen interactions, and as highlighted in our previous study (23), we stratified data into two VHH paratopes based on FR2 involvement and analyzed CDR3 length-dependent patterns within these subgroups (**Figure 6A**). The findings indicated that for VHHs utilizing FR2 in interactions, the correlation patterns persisted across VHH segments, although no correlation was observed with the overall interface size. For VHHs that did not involve FR2 in antigen interactions, the correlation patterns were preserved in CDR1 and CDR3 but were lost in CDR2. These results suggest that the correlation between interaction interface and CDR3 length is influenced by the types of VHH paratopes, and the involvement of FR2 in binding.

### 3.6 CDR3 length-dependent VHH/antigen interaction characteristics

**Figure 7:**
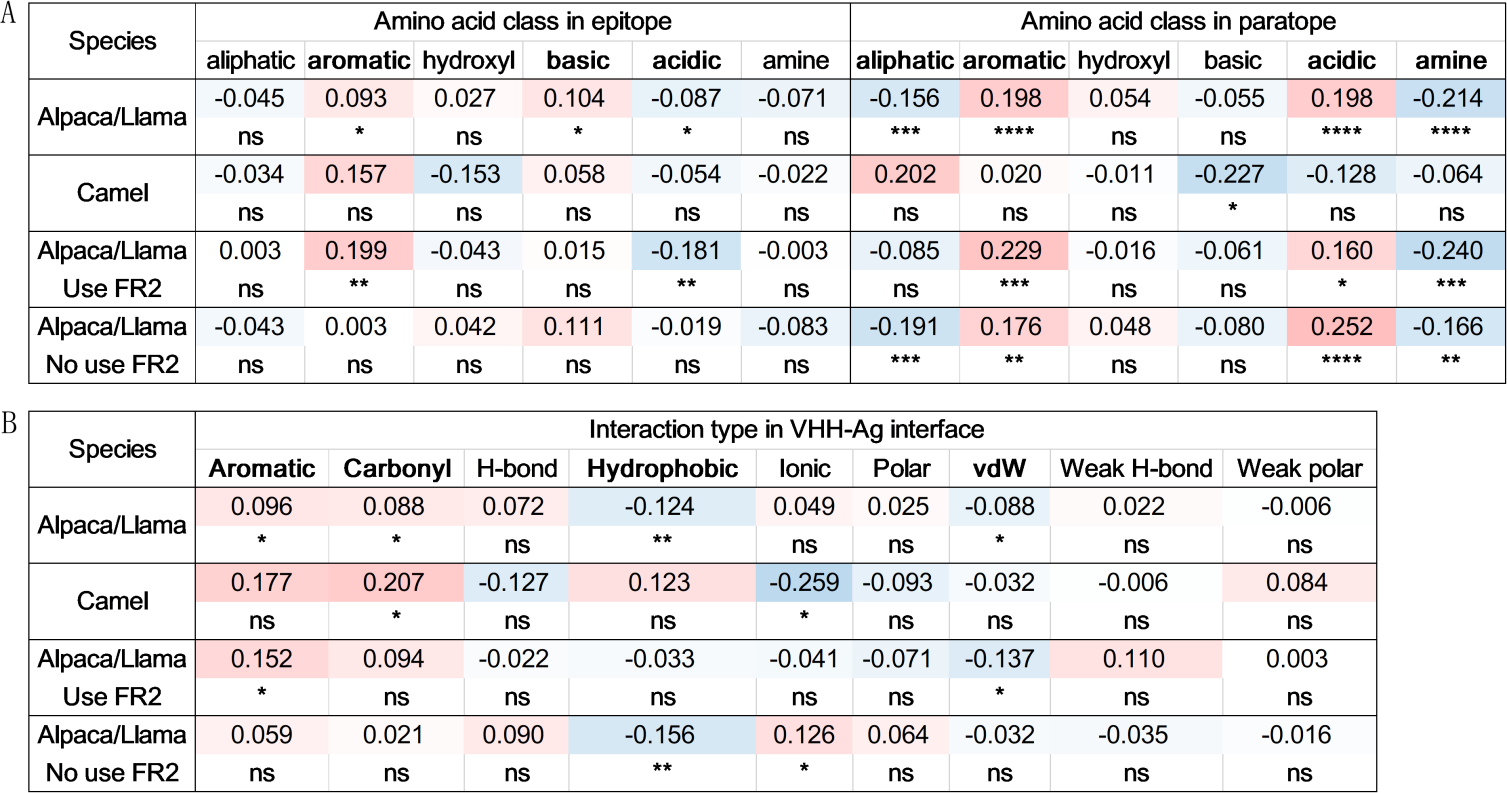
CDR3 length-dependent interaction patterns. Pearson correlations and significance indicators for the CDR3 length with the percentage of each amino acid class within epitopes or paratopes **(A)**, and percentage of interaction types in interface **(B)** across species and subtypes. Bold highlights indicate features with significant correlations in the Alpaca/Llama. P-values are marked as followings: ns: P > 0.05; *0.01< P <=0.05; **0.001 <= P < 0.01; ****P <= 0.0001.

To study possible CDR3 length-dependent interaction characteristics, we investigated the correlation between amino acid composition, interatomic interactions at the interface, and CDR3 length. The amino acid compositions of epitopes and paratopes were categorized into seven classes: aliphatic, aromatic, sulfur-containing, hydroxyl, basic, acidic, and amine. Despite the conserved amino acid compositions of epitopes and paratopes across species (**Supplementary Figures S11A, B**), distinct correlation patterns with CDR3 length were observed (**Figure 7A**). In Alpacas and Llamas, VHHs with longer CDR3 tend to utilize more aromatic and acidic amino acids in interactions, while the use of aliphatic and amine amino acids decreases. In contrast, targeted antigen epitopes are richer in aromatic and basic amino acids with longer CDR3. The negative correlation between aliphatic amino acids in the paratope and CDR3 length may be linked to the reduced surface hydrophobicity in VHHs with longer CDR3. Additionally, the increased presence of acidic amino acids aligns with findings that longer CDR3 VHHs contain more acidic residues and exhibit a lower charge. The decrease in amine usage may result from reduced residue N usage at position 105 in the CDR3. These CDR3 length-related patterns are present in the two types of VHH paratopes but are not observed in camel VHHs.

Analysis of interatomic interactions shows a high degree of consistency in interaction types across species (**Supplementary Figure S11C**), with hydrophobic interactions being predominant. However, distinct correlation patterns with CDR3 length were observed (**Figure 7B**). In Alpacas and Llamas, CDR3 length is positively correlated with aromatic interactions and negatively with hydrophobic interactions. These results align with the amino acid composition patterns in epitopes and paratopes. There is also a positive correlation with carbonyl interactions and a negative correlation with van der Waals interactions. Within subdivided VHH paratope subgroups, some of these patterns remain consistent. However, in VHHs from Camels, different patterns emerge compared to VHHs from Alpacas and Llamas. There is a slightly positive correlation between CDR3 length and hydrophobic interactions, corresponding with increased aliphatic amino acid usage, and a significant negative correlation between CDR3 length and ionic interactions. These findings collectively demonstrate species-specific CDR3 length-dependent interaction patterns.

## 4 Discussions

A typical VHH sequence, excluding CDR3, spans approximately 100–105 amino acids. CDR3 length varies significantly, ranging from as short as 5 amino acids (about 5% of total VHH sequence) to 25 or more amino acids (over 20% of total sequence). The total length difference can be as large as 20% between VHHs with short and long CDR3s. It is unsurprising to observe significant differences in sequence and structural characteristics between VHHs with short and long CDR3 regions. In this study, we systematically analyzed the correlations between CDR3 length and various VHH sequence and structural features, and VHH/antigen interaction characteristics. Overall, we found that longer CDR3 are associated with VHHs that exhibit lower net charge, reduced surface charge, lower surface hydrophobicity, larger surface area, more interactions between CDR3 and other parts of VHHs, more likely to have bent conformation for CDR3, more 310 helix and less β-sheet secondary structures in CDR3, and less contribution from CDR1/CDR2/FR2 in antigen binding. We observed more pronounced CDR3 length-dependent patterns in VHHs from Alpaca and Llama as compared to those from Camel. This may not be surprising, as VHHs from Alpaca and Llama display broader range of CDR3 length distribution compared to VHHs from Bactrian. These differences may reflect distinct evolutionary pressures and functional adaptations in the immune repertoires of these species. The wider CDR3 length distribution in VHHs from Alpaca and Llama may compensate for the diversity loss from low proportion of VHHs in Alpaca and Llama repertoires (1).

Consistent with our previous study (23), there are certain preferences between CDR3 length and the germline usages. For example, IGHV3S53*01 is predominantly used by VHHs with short CDR3, while IGHV3S65*01 is more commonly found in VHHs with long CDR3. These findings help explain some of the correlations observed in the study. However, the CDR3 length-dependent patterns of CDR3-related properties cannot be fully attributed to the germlines used in the VHH, as CDR3 is the result of VDJ recombination. During VHH B cell development, selection pressures may shape the immune repertoire, resulting in the specific CDR3 length-dependent patterns observed in this study.

Using the C-terminal stem of CDR3, Bahrami et al. (40) classified CDR3 in VHHs into extended and kinked conformations. Aided by visual inspection, Kuroda and Tsumoto (41) grouped CDR3 conformations into three structural classes: helical bending, kinked, and extended. In our study, we applied a simple distance metric (FR2_CDR3_Dis) to classify CDR3 conformations into two groups, as evident by the two distinct peaks in the distance metric density plot (**Figure 5A**). Interestingly, the density plot for VHHs from Camel shifted slightly to the left compared to the density plot for VHHs from Alpaca/Llama. These results suggest that the criteria for separating CDR3 conformations for VHHs from different species may need to be slightly adjusted. The observed negative correlation between the distance metric and CDR3 length (**Figures 4E**, **5E**), along with the fact that VHHs from Camel tend to have longer CDR3 than those from Alpaca/Llama (**Table 1**), may explain the left shift in the density plot.

The structural analysis reveals that CDR3 length plays a key role in shaping the conformation and interaction patterns of VHHs. Longer CDR3s tend to adopt a bent conformation, bringing them closer to the FR2 and CDR2 regions, while shorter CDR3s are more likely to extend away from these regions. This conformational difference is reflected in the increased number of interactions between longer CDR3s and other segments of the VHH, especially with CDR2 and FR2. These interactions may contribute to the stability and antigen-binding properties of VHHs with longer CDR3s. The secondary structure analysis further supports the idea that CDR3 length influences the structural flexibility of VHHs. Longer CDR3s are associated with increased usage of helices and coils, while shorter CDR3s favor β-sheets and turns. These structural preferences may reflect the need for longer CDR3s to adopt more flexible conformations to interact effectively with antigens. However, more studies will be needed to confirm some of those observations as structure data set used in the analysis is quite small.

To analyze the impact of antibody numbering schemes on CDR3 length dependent patterns found in this study, same analyses were performed using Chothia numbering (44), which is a structure-based scheme. Similar significant correlations between CDR3 length and VHH charge/hydropathy, CDR3 interaction with other VHH fragments, CDR3 second structures, number of antigen contact residues in VHH, CDR1/2/3, FR2 and amino acid usages in FR2 were found (**Supplementary Figure S13**).

In this study, we did not include an analysis of NGS data for VHHs from Dromedary, as we were unable to access such a dataset. Based on germlines analysis, we expect that VHHs from Dromedary will be more similar to those from Bactrian than to those from Alpaca/Llama. Indeed, UMAP clustering of VHHs sequences from structure dataset showed that many sequences from Dromedary and Bactrian clustered together, while sequences from Alpaca and Llama formed separate clusters (**Supplementary Figure S12**). Many of CDR3 length dependent patterns found in this study appear to be VHH specific as similar analyses on human VH sequences showed quite distinct results especially in amino acid usages of FR2 region (**Supplementary Figure S14**). There is no correlation found between CDR3 length and amino acid usages in FR2 from human VH.

The findings of this study have significant implications for the development of therapeutic VHHs. The distinct CDR3 length-dependent sequence and structural patterns observed in VHHs from different species suggest that these properties can be tailored to optimize antibody specificity, stability, and antigen-binding affinity. For example, the increased negative charge associated with longer CDR3 regions may enhance the solubility and specificity of therapeutic VHHs, while the conformational flexibility of longer CDR3 regions may improve their ability to bind to complex or hidden epitopes. Furthermore, the species-specific differences in CDR3 length and its impact on VHH properties highlight the potential of leveraging the unique characteristics of VHHs from different species for therapeutic applications. For instance, the longer and more narrowly distributed CDR3 regions in Bactrian VHHs may be particularly well-suited for targeting specific antigens, while the broader CDR3 length distribution in Alpaca and Llama VHHs may offer greater diversity in antigen recognition. In addition, CDR3 length-dependent features discovered in this study can help to design more natural VHH sequences. For example, to design VHHs with long CDR3, we may want to use F at IMGT 42 position and more negative CDR3 sequences while for VHHs with short CDR3, we may want to use Y at IMGT 42 position and neutral or less negative CDR3 sequences.

## 5 Conclusion

This study provides valuable insights into the sequence, structural, and functional characteristics associated with CDR3 length in VHHs. These findings not only deepen our understanding of the molecular mechanisms underlying antibody-antigen interactions but also offer practical guidance for the rational design of therapeutic VHHs with optimized properties for specific applications. Future studies could further explore the functional implications of CDR3 length *in vivo* and investigate the potential for engineering VHHs with tailored CDR3 lengths to achieve desired therapeutic outcomes.

## Data Availability

Raw sequencing data generated from this study were submitted to Sequence Read Archive (SRA) under accession number PRJNA1148326 for Alpaca, PRJNA1291406 for Llama and Bactrian.
