# Supplementary material for: Comparative analysis of CDR3 length-dependent patterns in VHHs
Source: Front Immunol. 2025 Aug 15;16:1647230. doi: 10.3389/fimmu.2025.1647230 (PMC12394203; doi:10.3389/fimmu.2025.1647230)
Supplement: Supplementary file 1 [file Presentation1.pptx]

## Slide 1
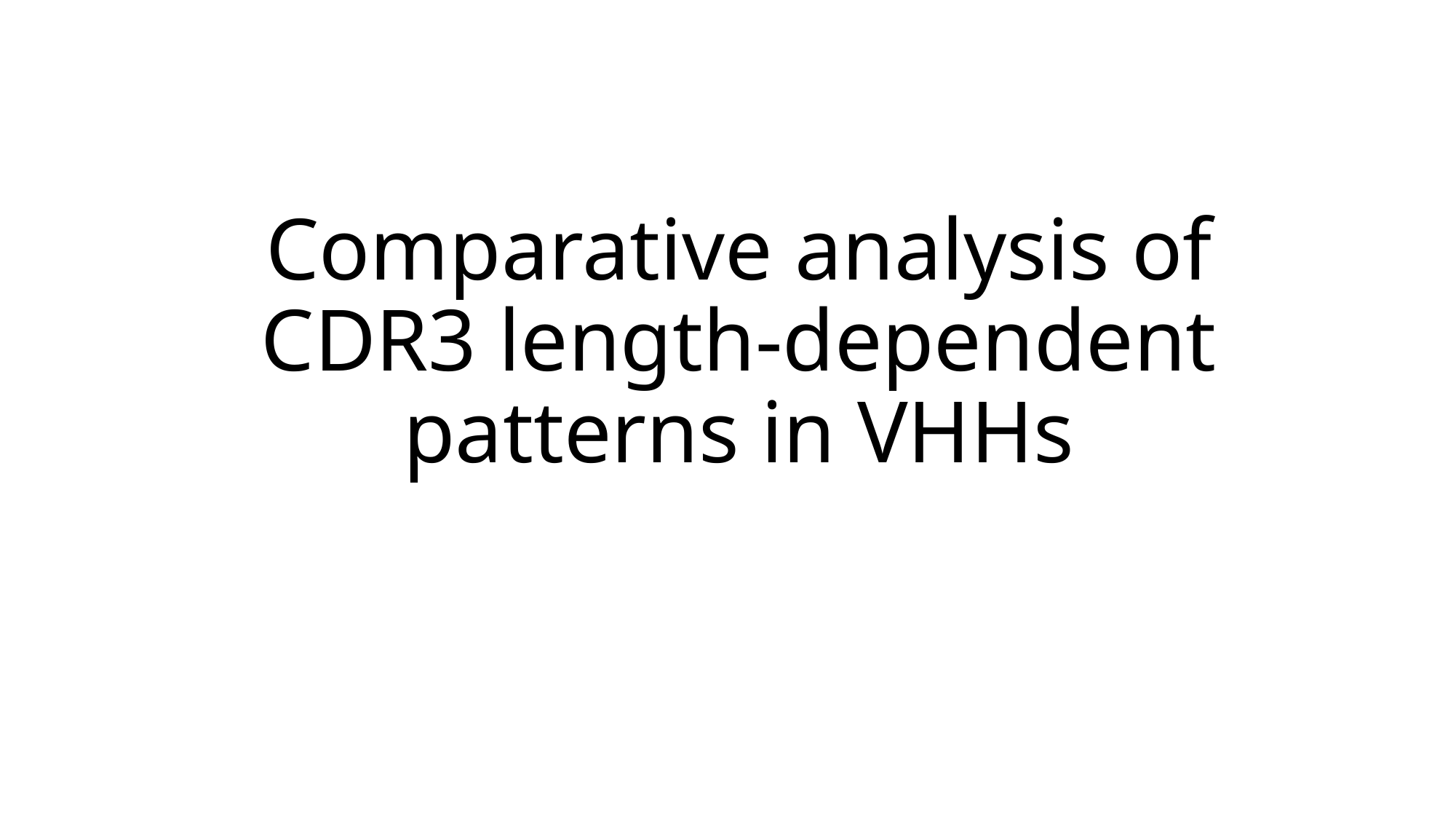

# Comparative analysis of CDR3 length-dependent patterns in VHHs

## Slide 2
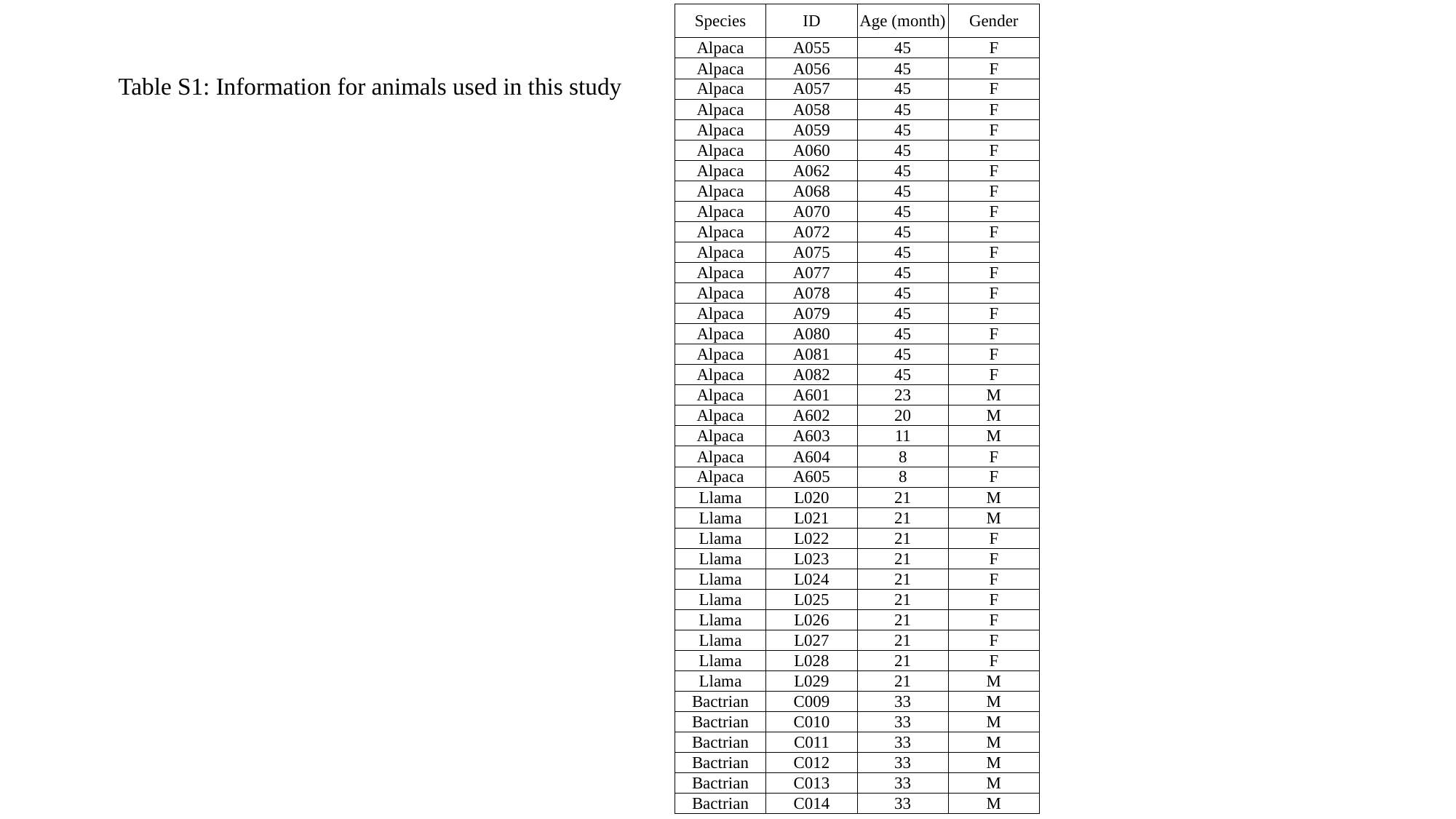

| Species | ID | Age (month) | Gender |
| --- | --- | --- | --- |
| Alpaca | A055 | 45 | F |
| Alpaca | A056 | 45 | F |
| Alpaca | A057 | 45 | F |
| Alpaca | A058 | 45 | F |
| Alpaca | A059 | 45 | F |
| Alpaca | A060 | 45 | F |
| Alpaca | A062 | 45 | F |
| Alpaca | A068 | 45 | F |
| Alpaca | A070 | 45 | F |
| Alpaca | A072 | 45 | F |
| Alpaca | A075 | 45 | F |
| Alpaca | A077 | 45 | F |
| Alpaca | A078 | 45 | F |
| Alpaca | A079 | 45 | F |
| Alpaca | A080 | 45 | F |
| Alpaca | A081 | 45 | F |
| Alpaca | A082 | 45 | F |
| Alpaca | A601 | 23 | M |
| Alpaca | A602 | 20 | M |
| Alpaca | A603 | 11 | M |
| Alpaca | A604 | 8 | F |
| Alpaca | A605 | 8 | F |
| Llama | L020 | 21 | M |
| Llama | L021 | 21 | M |
| Llama | L022 | 21 | F |
| Llama | L023 | 21 | F |
| Llama | L024 | 21 | F |
| Llama | L025 | 21 | F |
| Llama | L026 | 21 | F |
| Llama | L027 | 21 | F |
| Llama | L028 | 21 | F |
| Llama | L029 | 21 | M |
| Bactrian | C009 | 33 | M |
| Bactrian | C010 | 33 | M |
| Bactrian | C011 | 33 | M |
| Bactrian | C012 | 33 | M |
| Bactrian | C013 | 33 | M |
| Bactrian | C014 | 33 | M |
Table S1: Information for animals used in this study

## Slide 3
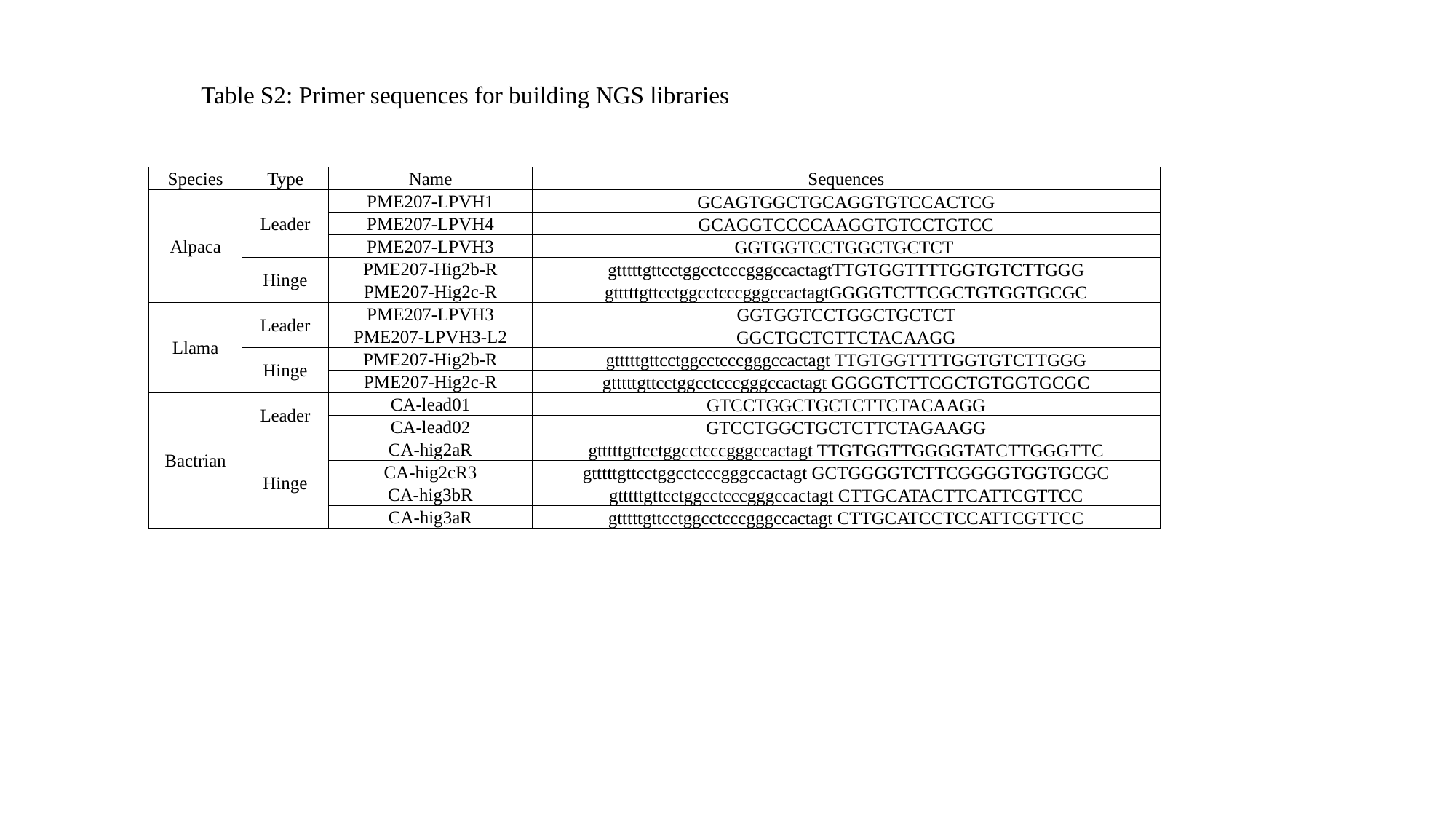

Table S2: Primer sequences for building NGS libraries
| Species | Type | Name | Sequences |
| --- | --- | --- | --- |
| Alpaca | Leader | PME207-LPVH1 | GCAGTGGCTGCAGGTGTCCACTCG |
| | | PME207-LPVH4 | GCAGGTCCCCAAGGTGTCCTGTCC |
| | | PME207-LPVH3 | GGTGGTCCTGGCTGCTCT |
| | Hinge | PME207-Hig2b-R | gtttttgttcctggcctcccgggccactagtTTGTGGTTTTGGTGTCTTGGG |
| | | PME207-Hig2c-R | gtttttgttcctggcctcccgggccactagtGGGGTCTTCGCTGTGGTGCGC |
| Llama | Leader | PME207-LPVH3 | GGTGGTCCTGGCTGCTCT |
| | | PME207-LPVH3-L2 | GGCTGCTCTTCTACAAGG |
| | Hinge | PME207-Hig2b-R | gtttttgttcctggcctcccgggccactagt TTGTGGTTTTGGTGTCTTGGG |
| | | PME207-Hig2c-R | gtttttgttcctggcctcccgggccactagt GGGGTCTTCGCTGTGGTGCGC |
| Bactrian | Leader | CA-lead01 | GTCCTGGCTGCTCTTCTACAAGG |
| | | CA-lead02 | GTCCTGGCTGCTCTTCTAGAAGG |
| | Hinge | CA-hig2aR | gtttttgttcctggcctcccgggccactagt TTGTGGTTGGGGTATCTTGGGTTC |
| | | CA-hig2cR3 | gtttttgttcctggcctcccgggccactagt GCTGGGGTCTTCGGGGTGGTGCGC |
| | | CA-hig3bR | gtttttgttcctggcctcccgggccactagt CTTGCATACTTCATTCGTTCC |
| | | CA-hig3aR | gtttttgttcctggcctcccgggccactagt CTTGCATCCTCCATTCGTTCC |

## Slide 4
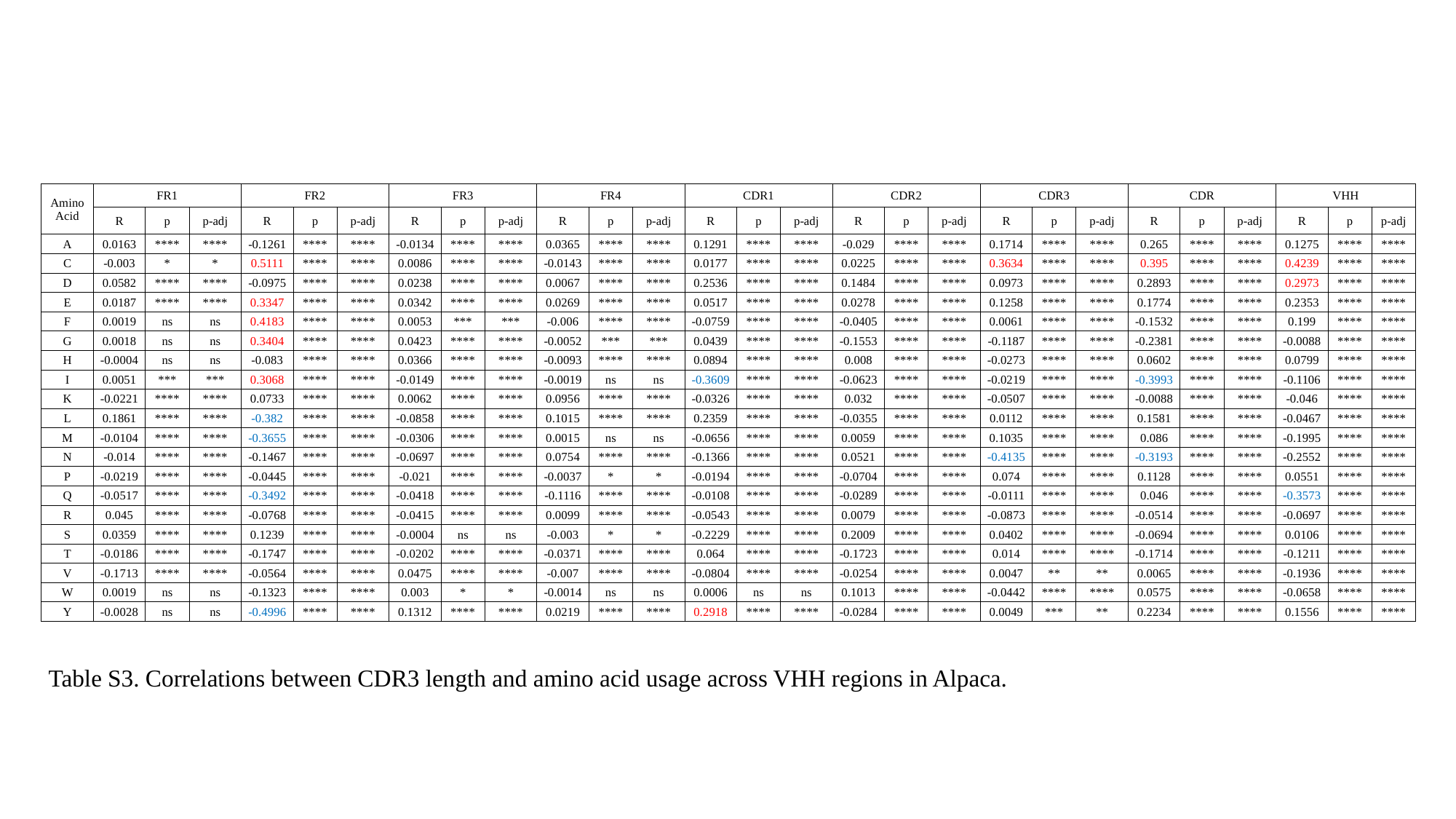

| Amino Acid | FR1 | | | FR2 | | | FR3 | | | FR4 | | | CDR1 | | | CDR2 | | | CDR3 | | | CDR | | | VHH | | |
| --- | --- | --- | --- | --- | --- | --- | --- | --- | --- | --- | --- | --- | --- | --- | --- | --- | --- | --- | --- | --- | --- | --- | --- | --- | --- | --- | --- |
| | R | p | p-adj | R | p | p-adj | R | p | p-adj | R | p | p-adj | R | p | p-adj | R | p | p-adj | R | p | p-adj | R | p | p-adj | R | p | p-adj |
| A | 0.0163 | \*\*\*\* | \*\*\*\* | -0.1261 | \*\*\*\* | \*\*\*\* | -0.0134 | \*\*\*\* | \*\*\*\* | 0.0365 | \*\*\*\* | \*\*\*\* | 0.1291 | \*\*\*\* | \*\*\*\* | -0.029 | \*\*\*\* | \*\*\*\* | 0.1714 | \*\*\*\* | \*\*\*\* | 0.265 | \*\*\*\* | \*\*\*\* | 0.1275 | \*\*\*\* | \*\*\*\* |
| C | -0.003 | \* | \* | 0.5111 | \*\*\*\* | \*\*\*\* | 0.0086 | \*\*\*\* | \*\*\*\* | -0.0143 | \*\*\*\* | \*\*\*\* | 0.0177 | \*\*\*\* | \*\*\*\* | 0.0225 | \*\*\*\* | \*\*\*\* | 0.3634 | \*\*\*\* | \*\*\*\* | 0.395 | \*\*\*\* | \*\*\*\* | 0.4239 | \*\*\*\* | \*\*\*\* |
| D | 0.0582 | \*\*\*\* | \*\*\*\* | -0.0975 | \*\*\*\* | \*\*\*\* | 0.0238 | \*\*\*\* | \*\*\*\* | 0.0067 | \*\*\*\* | \*\*\*\* | 0.2536 | \*\*\*\* | \*\*\*\* | 0.1484 | \*\*\*\* | \*\*\*\* | 0.0973 | \*\*\*\* | \*\*\*\* | 0.2893 | \*\*\*\* | \*\*\*\* | 0.2973 | \*\*\*\* | \*\*\*\* |
| E | 0.0187 | \*\*\*\* | \*\*\*\* | 0.3347 | \*\*\*\* | \*\*\*\* | 0.0342 | \*\*\*\* | \*\*\*\* | 0.0269 | \*\*\*\* | \*\*\*\* | 0.0517 | \*\*\*\* | \*\*\*\* | 0.0278 | \*\*\*\* | \*\*\*\* | 0.1258 | \*\*\*\* | \*\*\*\* | 0.1774 | \*\*\*\* | \*\*\*\* | 0.2353 | \*\*\*\* | \*\*\*\* |
| F | 0.0019 | ns | ns | 0.4183 | \*\*\*\* | \*\*\*\* | 0.0053 | \*\*\* | \*\*\* | -0.006 | \*\*\*\* | \*\*\*\* | -0.0759 | \*\*\*\* | \*\*\*\* | -0.0405 | \*\*\*\* | \*\*\*\* | 0.0061 | \*\*\*\* | \*\*\*\* | -0.1532 | \*\*\*\* | \*\*\*\* | 0.199 | \*\*\*\* | \*\*\*\* |
| G | 0.0018 | ns | ns | 0.3404 | \*\*\*\* | \*\*\*\* | 0.0423 | \*\*\*\* | \*\*\*\* | -0.0052 | \*\*\* | \*\*\* | 0.0439 | \*\*\*\* | \*\*\*\* | -0.1553 | \*\*\*\* | \*\*\*\* | -0.1187 | \*\*\*\* | \*\*\*\* | -0.2381 | \*\*\*\* | \*\*\*\* | -0.0088 | \*\*\*\* | \*\*\*\* |
| H | -0.0004 | ns | ns | -0.083 | \*\*\*\* | \*\*\*\* | 0.0366 | \*\*\*\* | \*\*\*\* | -0.0093 | \*\*\*\* | \*\*\*\* | 0.0894 | \*\*\*\* | \*\*\*\* | 0.008 | \*\*\*\* | \*\*\*\* | -0.0273 | \*\*\*\* | \*\*\*\* | 0.0602 | \*\*\*\* | \*\*\*\* | 0.0799 | \*\*\*\* | \*\*\*\* |
| I | 0.0051 | \*\*\* | \*\*\* | 0.3068 | \*\*\*\* | \*\*\*\* | -0.0149 | \*\*\*\* | \*\*\*\* | -0.0019 | ns | ns | -0.3609 | \*\*\*\* | \*\*\*\* | -0.0623 | \*\*\*\* | \*\*\*\* | -0.0219 | \*\*\*\* | \*\*\*\* | -0.3993 | \*\*\*\* | \*\*\*\* | -0.1106 | \*\*\*\* | \*\*\*\* |
| K | -0.0221 | \*\*\*\* | \*\*\*\* | 0.0733 | \*\*\*\* | \*\*\*\* | 0.0062 | \*\*\*\* | \*\*\*\* | 0.0956 | \*\*\*\* | \*\*\*\* | -0.0326 | \*\*\*\* | \*\*\*\* | 0.032 | \*\*\*\* | \*\*\*\* | -0.0507 | \*\*\*\* | \*\*\*\* | -0.0088 | \*\*\*\* | \*\*\*\* | -0.046 | \*\*\*\* | \*\*\*\* |
| L | 0.1861 | \*\*\*\* | \*\*\*\* | -0.382 | \*\*\*\* | \*\*\*\* | -0.0858 | \*\*\*\* | \*\*\*\* | 0.1015 | \*\*\*\* | \*\*\*\* | 0.2359 | \*\*\*\* | \*\*\*\* | -0.0355 | \*\*\*\* | \*\*\*\* | 0.0112 | \*\*\*\* | \*\*\*\* | 0.1581 | \*\*\*\* | \*\*\*\* | -0.0467 | \*\*\*\* | \*\*\*\* |
| M | -0.0104 | \*\*\*\* | \*\*\*\* | -0.3655 | \*\*\*\* | \*\*\*\* | -0.0306 | \*\*\*\* | \*\*\*\* | 0.0015 | ns | ns | -0.0656 | \*\*\*\* | \*\*\*\* | 0.0059 | \*\*\*\* | \*\*\*\* | 0.1035 | \*\*\*\* | \*\*\*\* | 0.086 | \*\*\*\* | \*\*\*\* | -0.1995 | \*\*\*\* | \*\*\*\* |
| N | -0.014 | \*\*\*\* | \*\*\*\* | -0.1467 | \*\*\*\* | \*\*\*\* | -0.0697 | \*\*\*\* | \*\*\*\* | 0.0754 | \*\*\*\* | \*\*\*\* | -0.1366 | \*\*\*\* | \*\*\*\* | 0.0521 | \*\*\*\* | \*\*\*\* | -0.4135 | \*\*\*\* | \*\*\*\* | -0.3193 | \*\*\*\* | \*\*\*\* | -0.2552 | \*\*\*\* | \*\*\*\* |
| P | -0.0219 | \*\*\*\* | \*\*\*\* | -0.0445 | \*\*\*\* | \*\*\*\* | -0.021 | \*\*\*\* | \*\*\*\* | -0.0037 | \* | \* | -0.0194 | \*\*\*\* | \*\*\*\* | -0.0704 | \*\*\*\* | \*\*\*\* | 0.074 | \*\*\*\* | \*\*\*\* | 0.1128 | \*\*\*\* | \*\*\*\* | 0.0551 | \*\*\*\* | \*\*\*\* |
| Q | -0.0517 | \*\*\*\* | \*\*\*\* | -0.3492 | \*\*\*\* | \*\*\*\* | -0.0418 | \*\*\*\* | \*\*\*\* | -0.1116 | \*\*\*\* | \*\*\*\* | -0.0108 | \*\*\*\* | \*\*\*\* | -0.0289 | \*\*\*\* | \*\*\*\* | -0.0111 | \*\*\*\* | \*\*\*\* | 0.046 | \*\*\*\* | \*\*\*\* | -0.3573 | \*\*\*\* | \*\*\*\* |
| R | 0.045 | \*\*\*\* | \*\*\*\* | -0.0768 | \*\*\*\* | \*\*\*\* | -0.0415 | \*\*\*\* | \*\*\*\* | 0.0099 | \*\*\*\* | \*\*\*\* | -0.0543 | \*\*\*\* | \*\*\*\* | 0.0079 | \*\*\*\* | \*\*\*\* | -0.0873 | \*\*\*\* | \*\*\*\* | -0.0514 | \*\*\*\* | \*\*\*\* | -0.0697 | \*\*\*\* | \*\*\*\* |
| S | 0.0359 | \*\*\*\* | \*\*\*\* | 0.1239 | \*\*\*\* | \*\*\*\* | -0.0004 | ns | ns | -0.003 | \* | \* | -0.2229 | \*\*\*\* | \*\*\*\* | 0.2009 | \*\*\*\* | \*\*\*\* | 0.0402 | \*\*\*\* | \*\*\*\* | -0.0694 | \*\*\*\* | \*\*\*\* | 0.0106 | \*\*\*\* | \*\*\*\* |
| T | -0.0186 | \*\*\*\* | \*\*\*\* | -0.1747 | \*\*\*\* | \*\*\*\* | -0.0202 | \*\*\*\* | \*\*\*\* | -0.0371 | \*\*\*\* | \*\*\*\* | 0.064 | \*\*\*\* | \*\*\*\* | -0.1723 | \*\*\*\* | \*\*\*\* | 0.014 | \*\*\*\* | \*\*\*\* | -0.1714 | \*\*\*\* | \*\*\*\* | -0.1211 | \*\*\*\* | \*\*\*\* |
| V | -0.1713 | \*\*\*\* | \*\*\*\* | -0.0564 | \*\*\*\* | \*\*\*\* | 0.0475 | \*\*\*\* | \*\*\*\* | -0.007 | \*\*\*\* | \*\*\*\* | -0.0804 | \*\*\*\* | \*\*\*\* | -0.0254 | \*\*\*\* | \*\*\*\* | 0.0047 | \*\* | \*\* | 0.0065 | \*\*\*\* | \*\*\*\* | -0.1936 | \*\*\*\* | \*\*\*\* |
| W | 0.0019 | ns | ns | -0.1323 | \*\*\*\* | \*\*\*\* | 0.003 | \* | \* | -0.0014 | ns | ns | 0.0006 | ns | ns | 0.1013 | \*\*\*\* | \*\*\*\* | -0.0442 | \*\*\*\* | \*\*\*\* | 0.0575 | \*\*\*\* | \*\*\*\* | -0.0658 | \*\*\*\* | \*\*\*\* |
| Y | -0.0028 | ns | ns | -0.4996 | \*\*\*\* | \*\*\*\* | 0.1312 | \*\*\*\* | \*\*\*\* | 0.0219 | \*\*\*\* | \*\*\*\* | 0.2918 | \*\*\*\* | \*\*\*\* | -0.0284 | \*\*\*\* | \*\*\*\* | 0.0049 | \*\*\* | \*\* | 0.2234 | \*\*\*\* | \*\*\*\* | 0.1556 | \*\*\*\* | \*\*\*\* |
Table S3. Correlations between CDR3 length and amino acid usage across VHH regions in Alpaca.

## Slide 5
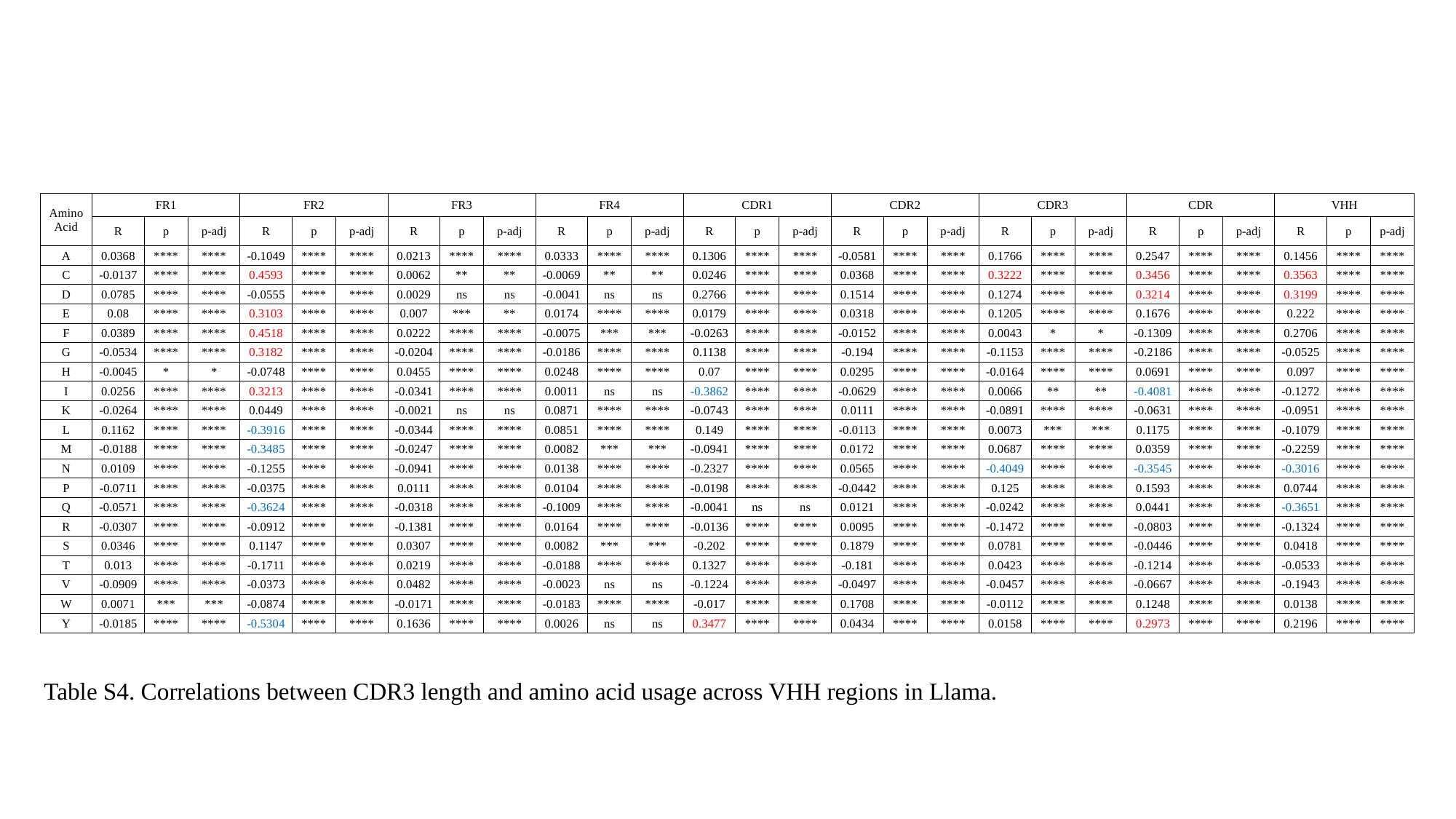

| Amino Acid | FR1 | | | FR2 | | | FR3 | | | FR4 | | | CDR1 | | | CDR2 | | | CDR3 | | | CDR | | | VHH | | |
| --- | --- | --- | --- | --- | --- | --- | --- | --- | --- | --- | --- | --- | --- | --- | --- | --- | --- | --- | --- | --- | --- | --- | --- | --- | --- | --- | --- |
| | R | p | p-adj | R | p | p-adj | R | p | p-adj | R | p | p-adj | R | p | p-adj | R | p | p-adj | R | p | p-adj | R | p | p-adj | R | p | p-adj |
| A | 0.0368 | \*\*\*\* | \*\*\*\* | -0.1049 | \*\*\*\* | \*\*\*\* | 0.0213 | \*\*\*\* | \*\*\*\* | 0.0333 | \*\*\*\* | \*\*\*\* | 0.1306 | \*\*\*\* | \*\*\*\* | -0.0581 | \*\*\*\* | \*\*\*\* | 0.1766 | \*\*\*\* | \*\*\*\* | 0.2547 | \*\*\*\* | \*\*\*\* | 0.1456 | \*\*\*\* | \*\*\*\* |
| C | -0.0137 | \*\*\*\* | \*\*\*\* | 0.4593 | \*\*\*\* | \*\*\*\* | 0.0062 | \*\* | \*\* | -0.0069 | \*\* | \*\* | 0.0246 | \*\*\*\* | \*\*\*\* | 0.0368 | \*\*\*\* | \*\*\*\* | 0.3222 | \*\*\*\* | \*\*\*\* | 0.3456 | \*\*\*\* | \*\*\*\* | 0.3563 | \*\*\*\* | \*\*\*\* |
| D | 0.0785 | \*\*\*\* | \*\*\*\* | -0.0555 | \*\*\*\* | \*\*\*\* | 0.0029 | ns | ns | -0.0041 | ns | ns | 0.2766 | \*\*\*\* | \*\*\*\* | 0.1514 | \*\*\*\* | \*\*\*\* | 0.1274 | \*\*\*\* | \*\*\*\* | 0.3214 | \*\*\*\* | \*\*\*\* | 0.3199 | \*\*\*\* | \*\*\*\* |
| E | 0.08 | \*\*\*\* | \*\*\*\* | 0.3103 | \*\*\*\* | \*\*\*\* | 0.007 | \*\*\* | \*\* | 0.0174 | \*\*\*\* | \*\*\*\* | 0.0179 | \*\*\*\* | \*\*\*\* | 0.0318 | \*\*\*\* | \*\*\*\* | 0.1205 | \*\*\*\* | \*\*\*\* | 0.1676 | \*\*\*\* | \*\*\*\* | 0.222 | \*\*\*\* | \*\*\*\* |
| F | 0.0389 | \*\*\*\* | \*\*\*\* | 0.4518 | \*\*\*\* | \*\*\*\* | 0.0222 | \*\*\*\* | \*\*\*\* | -0.0075 | \*\*\* | \*\*\* | -0.0263 | \*\*\*\* | \*\*\*\* | -0.0152 | \*\*\*\* | \*\*\*\* | 0.0043 | \* | \* | -0.1309 | \*\*\*\* | \*\*\*\* | 0.2706 | \*\*\*\* | \*\*\*\* |
| G | -0.0534 | \*\*\*\* | \*\*\*\* | 0.3182 | \*\*\*\* | \*\*\*\* | -0.0204 | \*\*\*\* | \*\*\*\* | -0.0186 | \*\*\*\* | \*\*\*\* | 0.1138 | \*\*\*\* | \*\*\*\* | -0.194 | \*\*\*\* | \*\*\*\* | -0.1153 | \*\*\*\* | \*\*\*\* | -0.2186 | \*\*\*\* | \*\*\*\* | -0.0525 | \*\*\*\* | \*\*\*\* |
| H | -0.0045 | \* | \* | -0.0748 | \*\*\*\* | \*\*\*\* | 0.0455 | \*\*\*\* | \*\*\*\* | 0.0248 | \*\*\*\* | \*\*\*\* | 0.07 | \*\*\*\* | \*\*\*\* | 0.0295 | \*\*\*\* | \*\*\*\* | -0.0164 | \*\*\*\* | \*\*\*\* | 0.0691 | \*\*\*\* | \*\*\*\* | 0.097 | \*\*\*\* | \*\*\*\* |
| I | 0.0256 | \*\*\*\* | \*\*\*\* | 0.3213 | \*\*\*\* | \*\*\*\* | -0.0341 | \*\*\*\* | \*\*\*\* | 0.0011 | ns | ns | -0.3862 | \*\*\*\* | \*\*\*\* | -0.0629 | \*\*\*\* | \*\*\*\* | 0.0066 | \*\* | \*\* | -0.4081 | \*\*\*\* | \*\*\*\* | -0.1272 | \*\*\*\* | \*\*\*\* |
| K | -0.0264 | \*\*\*\* | \*\*\*\* | 0.0449 | \*\*\*\* | \*\*\*\* | -0.0021 | ns | ns | 0.0871 | \*\*\*\* | \*\*\*\* | -0.0743 | \*\*\*\* | \*\*\*\* | 0.0111 | \*\*\*\* | \*\*\*\* | -0.0891 | \*\*\*\* | \*\*\*\* | -0.0631 | \*\*\*\* | \*\*\*\* | -0.0951 | \*\*\*\* | \*\*\*\* |
| L | 0.1162 | \*\*\*\* | \*\*\*\* | -0.3916 | \*\*\*\* | \*\*\*\* | -0.0344 | \*\*\*\* | \*\*\*\* | 0.0851 | \*\*\*\* | \*\*\*\* | 0.149 | \*\*\*\* | \*\*\*\* | -0.0113 | \*\*\*\* | \*\*\*\* | 0.0073 | \*\*\* | \*\*\* | 0.1175 | \*\*\*\* | \*\*\*\* | -0.1079 | \*\*\*\* | \*\*\*\* |
| M | -0.0188 | \*\*\*\* | \*\*\*\* | -0.3485 | \*\*\*\* | \*\*\*\* | -0.0247 | \*\*\*\* | \*\*\*\* | 0.0082 | \*\*\* | \*\*\* | -0.0941 | \*\*\*\* | \*\*\*\* | 0.0172 | \*\*\*\* | \*\*\*\* | 0.0687 | \*\*\*\* | \*\*\*\* | 0.0359 | \*\*\*\* | \*\*\*\* | -0.2259 | \*\*\*\* | \*\*\*\* |
| N | 0.0109 | \*\*\*\* | \*\*\*\* | -0.1255 | \*\*\*\* | \*\*\*\* | -0.0941 | \*\*\*\* | \*\*\*\* | 0.0138 | \*\*\*\* | \*\*\*\* | -0.2327 | \*\*\*\* | \*\*\*\* | 0.0565 | \*\*\*\* | \*\*\*\* | -0.4049 | \*\*\*\* | \*\*\*\* | -0.3545 | \*\*\*\* | \*\*\*\* | -0.3016 | \*\*\*\* | \*\*\*\* |
| P | -0.0711 | \*\*\*\* | \*\*\*\* | -0.0375 | \*\*\*\* | \*\*\*\* | 0.0111 | \*\*\*\* | \*\*\*\* | 0.0104 | \*\*\*\* | \*\*\*\* | -0.0198 | \*\*\*\* | \*\*\*\* | -0.0442 | \*\*\*\* | \*\*\*\* | 0.125 | \*\*\*\* | \*\*\*\* | 0.1593 | \*\*\*\* | \*\*\*\* | 0.0744 | \*\*\*\* | \*\*\*\* |
| Q | -0.0571 | \*\*\*\* | \*\*\*\* | -0.3624 | \*\*\*\* | \*\*\*\* | -0.0318 | \*\*\*\* | \*\*\*\* | -0.1009 | \*\*\*\* | \*\*\*\* | -0.0041 | ns | ns | 0.0121 | \*\*\*\* | \*\*\*\* | -0.0242 | \*\*\*\* | \*\*\*\* | 0.0441 | \*\*\*\* | \*\*\*\* | -0.3651 | \*\*\*\* | \*\*\*\* |
| R | -0.0307 | \*\*\*\* | \*\*\*\* | -0.0912 | \*\*\*\* | \*\*\*\* | -0.1381 | \*\*\*\* | \*\*\*\* | 0.0164 | \*\*\*\* | \*\*\*\* | -0.0136 | \*\*\*\* | \*\*\*\* | 0.0095 | \*\*\*\* | \*\*\*\* | -0.1472 | \*\*\*\* | \*\*\*\* | -0.0803 | \*\*\*\* | \*\*\*\* | -0.1324 | \*\*\*\* | \*\*\*\* |
| S | 0.0346 | \*\*\*\* | \*\*\*\* | 0.1147 | \*\*\*\* | \*\*\*\* | 0.0307 | \*\*\*\* | \*\*\*\* | 0.0082 | \*\*\* | \*\*\* | -0.202 | \*\*\*\* | \*\*\*\* | 0.1879 | \*\*\*\* | \*\*\*\* | 0.0781 | \*\*\*\* | \*\*\*\* | -0.0446 | \*\*\*\* | \*\*\*\* | 0.0418 | \*\*\*\* | \*\*\*\* |
| T | 0.013 | \*\*\*\* | \*\*\*\* | -0.1711 | \*\*\*\* | \*\*\*\* | 0.0219 | \*\*\*\* | \*\*\*\* | -0.0188 | \*\*\*\* | \*\*\*\* | 0.1327 | \*\*\*\* | \*\*\*\* | -0.181 | \*\*\*\* | \*\*\*\* | 0.0423 | \*\*\*\* | \*\*\*\* | -0.1214 | \*\*\*\* | \*\*\*\* | -0.0533 | \*\*\*\* | \*\*\*\* |
| V | -0.0909 | \*\*\*\* | \*\*\*\* | -0.0373 | \*\*\*\* | \*\*\*\* | 0.0482 | \*\*\*\* | \*\*\*\* | -0.0023 | ns | ns | -0.1224 | \*\*\*\* | \*\*\*\* | -0.0497 | \*\*\*\* | \*\*\*\* | -0.0457 | \*\*\*\* | \*\*\*\* | -0.0667 | \*\*\*\* | \*\*\*\* | -0.1943 | \*\*\*\* | \*\*\*\* |
| W | 0.0071 | \*\*\* | \*\*\* | -0.0874 | \*\*\*\* | \*\*\*\* | -0.0171 | \*\*\*\* | \*\*\*\* | -0.0183 | \*\*\*\* | \*\*\*\* | -0.017 | \*\*\*\* | \*\*\*\* | 0.1708 | \*\*\*\* | \*\*\*\* | -0.0112 | \*\*\*\* | \*\*\*\* | 0.1248 | \*\*\*\* | \*\*\*\* | 0.0138 | \*\*\*\* | \*\*\*\* |
| Y | -0.0185 | \*\*\*\* | \*\*\*\* | -0.5304 | \*\*\*\* | \*\*\*\* | 0.1636 | \*\*\*\* | \*\*\*\* | 0.0026 | ns | ns | 0.3477 | \*\*\*\* | \*\*\*\* | 0.0434 | \*\*\*\* | \*\*\*\* | 0.0158 | \*\*\*\* | \*\*\*\* | 0.2973 | \*\*\*\* | \*\*\*\* | 0.2196 | \*\*\*\* | \*\*\*\* |
Table S4. Correlations between CDR3 length and amino acid usage across VHH regions in Llama.

## Slide 6
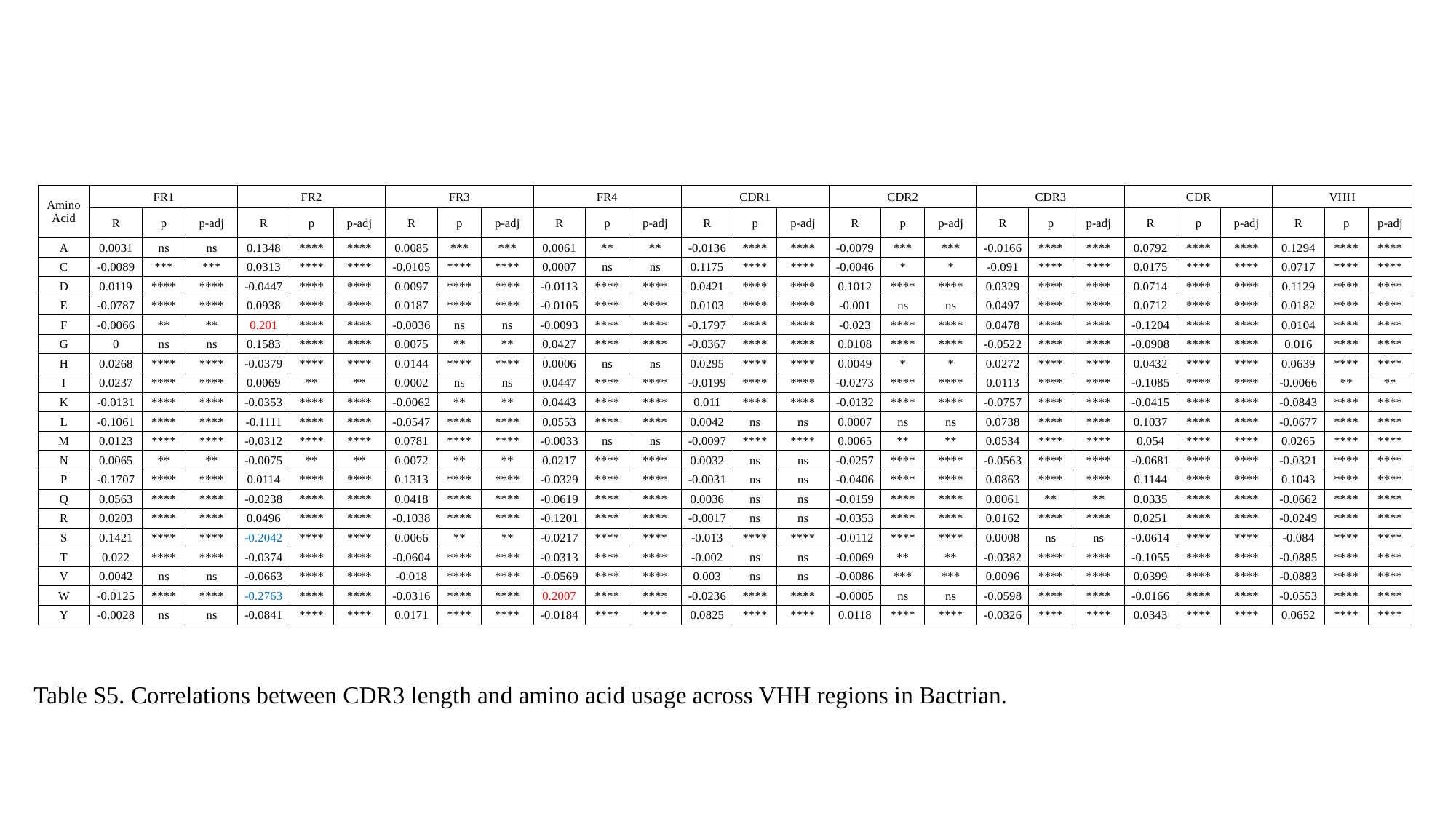

| Amino Acid | FR1 | | | FR2 | | | FR3 | | | FR4 | | | CDR1 | | | CDR2 | | | CDR3 | | | CDR | | | VHH | | |
| --- | --- | --- | --- | --- | --- | --- | --- | --- | --- | --- | --- | --- | --- | --- | --- | --- | --- | --- | --- | --- | --- | --- | --- | --- | --- | --- | --- |
| | R | p | p-adj | R | p | p-adj | R | p | p-adj | R | p | p-adj | R | p | p-adj | R | p | p-adj | R | p | p-adj | R | p | p-adj | R | p | p-adj |
| A | 0.0031 | ns | ns | 0.1348 | \*\*\*\* | \*\*\*\* | 0.0085 | \*\*\* | \*\*\* | 0.0061 | \*\* | \*\* | -0.0136 | \*\*\*\* | \*\*\*\* | -0.0079 | \*\*\* | \*\*\* | -0.0166 | \*\*\*\* | \*\*\*\* | 0.0792 | \*\*\*\* | \*\*\*\* | 0.1294 | \*\*\*\* | \*\*\*\* |
| C | -0.0089 | \*\*\* | \*\*\* | 0.0313 | \*\*\*\* | \*\*\*\* | -0.0105 | \*\*\*\* | \*\*\*\* | 0.0007 | ns | ns | 0.1175 | \*\*\*\* | \*\*\*\* | -0.0046 | \* | \* | -0.091 | \*\*\*\* | \*\*\*\* | 0.0175 | \*\*\*\* | \*\*\*\* | 0.0717 | \*\*\*\* | \*\*\*\* |
| D | 0.0119 | \*\*\*\* | \*\*\*\* | -0.0447 | \*\*\*\* | \*\*\*\* | 0.0097 | \*\*\*\* | \*\*\*\* | -0.0113 | \*\*\*\* | \*\*\*\* | 0.0421 | \*\*\*\* | \*\*\*\* | 0.1012 | \*\*\*\* | \*\*\*\* | 0.0329 | \*\*\*\* | \*\*\*\* | 0.0714 | \*\*\*\* | \*\*\*\* | 0.1129 | \*\*\*\* | \*\*\*\* |
| E | -0.0787 | \*\*\*\* | \*\*\*\* | 0.0938 | \*\*\*\* | \*\*\*\* | 0.0187 | \*\*\*\* | \*\*\*\* | -0.0105 | \*\*\*\* | \*\*\*\* | 0.0103 | \*\*\*\* | \*\*\*\* | -0.001 | ns | ns | 0.0497 | \*\*\*\* | \*\*\*\* | 0.0712 | \*\*\*\* | \*\*\*\* | 0.0182 | \*\*\*\* | \*\*\*\* |
| F | -0.0066 | \*\* | \*\* | 0.201 | \*\*\*\* | \*\*\*\* | -0.0036 | ns | ns | -0.0093 | \*\*\*\* | \*\*\*\* | -0.1797 | \*\*\*\* | \*\*\*\* | -0.023 | \*\*\*\* | \*\*\*\* | 0.0478 | \*\*\*\* | \*\*\*\* | -0.1204 | \*\*\*\* | \*\*\*\* | 0.0104 | \*\*\*\* | \*\*\*\* |
| G | 0 | ns | ns | 0.1583 | \*\*\*\* | \*\*\*\* | 0.0075 | \*\* | \*\* | 0.0427 | \*\*\*\* | \*\*\*\* | -0.0367 | \*\*\*\* | \*\*\*\* | 0.0108 | \*\*\*\* | \*\*\*\* | -0.0522 | \*\*\*\* | \*\*\*\* | -0.0908 | \*\*\*\* | \*\*\*\* | 0.016 | \*\*\*\* | \*\*\*\* |
| H | 0.0268 | \*\*\*\* | \*\*\*\* | -0.0379 | \*\*\*\* | \*\*\*\* | 0.0144 | \*\*\*\* | \*\*\*\* | 0.0006 | ns | ns | 0.0295 | \*\*\*\* | \*\*\*\* | 0.0049 | \* | \* | 0.0272 | \*\*\*\* | \*\*\*\* | 0.0432 | \*\*\*\* | \*\*\*\* | 0.0639 | \*\*\*\* | \*\*\*\* |
| I | 0.0237 | \*\*\*\* | \*\*\*\* | 0.0069 | \*\* | \*\* | 0.0002 | ns | ns | 0.0447 | \*\*\*\* | \*\*\*\* | -0.0199 | \*\*\*\* | \*\*\*\* | -0.0273 | \*\*\*\* | \*\*\*\* | 0.0113 | \*\*\*\* | \*\*\*\* | -0.1085 | \*\*\*\* | \*\*\*\* | -0.0066 | \*\* | \*\* |
| K | -0.0131 | \*\*\*\* | \*\*\*\* | -0.0353 | \*\*\*\* | \*\*\*\* | -0.0062 | \*\* | \*\* | 0.0443 | \*\*\*\* | \*\*\*\* | 0.011 | \*\*\*\* | \*\*\*\* | -0.0132 | \*\*\*\* | \*\*\*\* | -0.0757 | \*\*\*\* | \*\*\*\* | -0.0415 | \*\*\*\* | \*\*\*\* | -0.0843 | \*\*\*\* | \*\*\*\* |
| L | -0.1061 | \*\*\*\* | \*\*\*\* | -0.1111 | \*\*\*\* | \*\*\*\* | -0.0547 | \*\*\*\* | \*\*\*\* | 0.0553 | \*\*\*\* | \*\*\*\* | 0.0042 | ns | ns | 0.0007 | ns | ns | 0.0738 | \*\*\*\* | \*\*\*\* | 0.1037 | \*\*\*\* | \*\*\*\* | -0.0677 | \*\*\*\* | \*\*\*\* |
| M | 0.0123 | \*\*\*\* | \*\*\*\* | -0.0312 | \*\*\*\* | \*\*\*\* | 0.0781 | \*\*\*\* | \*\*\*\* | -0.0033 | ns | ns | -0.0097 | \*\*\*\* | \*\*\*\* | 0.0065 | \*\* | \*\* | 0.0534 | \*\*\*\* | \*\*\*\* | 0.054 | \*\*\*\* | \*\*\*\* | 0.0265 | \*\*\*\* | \*\*\*\* |
| N | 0.0065 | \*\* | \*\* | -0.0075 | \*\* | \*\* | 0.0072 | \*\* | \*\* | 0.0217 | \*\*\*\* | \*\*\*\* | 0.0032 | ns | ns | -0.0257 | \*\*\*\* | \*\*\*\* | -0.0563 | \*\*\*\* | \*\*\*\* | -0.0681 | \*\*\*\* | \*\*\*\* | -0.0321 | \*\*\*\* | \*\*\*\* |
| P | -0.1707 | \*\*\*\* | \*\*\*\* | 0.0114 | \*\*\*\* | \*\*\*\* | 0.1313 | \*\*\*\* | \*\*\*\* | -0.0329 | \*\*\*\* | \*\*\*\* | -0.0031 | ns | ns | -0.0406 | \*\*\*\* | \*\*\*\* | 0.0863 | \*\*\*\* | \*\*\*\* | 0.1144 | \*\*\*\* | \*\*\*\* | 0.1043 | \*\*\*\* | \*\*\*\* |
| Q | 0.0563 | \*\*\*\* | \*\*\*\* | -0.0238 | \*\*\*\* | \*\*\*\* | 0.0418 | \*\*\*\* | \*\*\*\* | -0.0619 | \*\*\*\* | \*\*\*\* | 0.0036 | ns | ns | -0.0159 | \*\*\*\* | \*\*\*\* | 0.0061 | \*\* | \*\* | 0.0335 | \*\*\*\* | \*\*\*\* | -0.0662 | \*\*\*\* | \*\*\*\* |
| R | 0.0203 | \*\*\*\* | \*\*\*\* | 0.0496 | \*\*\*\* | \*\*\*\* | -0.1038 | \*\*\*\* | \*\*\*\* | -0.1201 | \*\*\*\* | \*\*\*\* | -0.0017 | ns | ns | -0.0353 | \*\*\*\* | \*\*\*\* | 0.0162 | \*\*\*\* | \*\*\*\* | 0.0251 | \*\*\*\* | \*\*\*\* | -0.0249 | \*\*\*\* | \*\*\*\* |
| S | 0.1421 | \*\*\*\* | \*\*\*\* | -0.2042 | \*\*\*\* | \*\*\*\* | 0.0066 | \*\* | \*\* | -0.0217 | \*\*\*\* | \*\*\*\* | -0.013 | \*\*\*\* | \*\*\*\* | -0.0112 | \*\*\*\* | \*\*\*\* | 0.0008 | ns | ns | -0.0614 | \*\*\*\* | \*\*\*\* | -0.084 | \*\*\*\* | \*\*\*\* |
| T | 0.022 | \*\*\*\* | \*\*\*\* | -0.0374 | \*\*\*\* | \*\*\*\* | -0.0604 | \*\*\*\* | \*\*\*\* | -0.0313 | \*\*\*\* | \*\*\*\* | -0.002 | ns | ns | -0.0069 | \*\* | \*\* | -0.0382 | \*\*\*\* | \*\*\*\* | -0.1055 | \*\*\*\* | \*\*\*\* | -0.0885 | \*\*\*\* | \*\*\*\* |
| V | 0.0042 | ns | ns | -0.0663 | \*\*\*\* | \*\*\*\* | -0.018 | \*\*\*\* | \*\*\*\* | -0.0569 | \*\*\*\* | \*\*\*\* | 0.003 | ns | ns | -0.0086 | \*\*\* | \*\*\* | 0.0096 | \*\*\*\* | \*\*\*\* | 0.0399 | \*\*\*\* | \*\*\*\* | -0.0883 | \*\*\*\* | \*\*\*\* |
| W | -0.0125 | \*\*\*\* | \*\*\*\* | -0.2763 | \*\*\*\* | \*\*\*\* | -0.0316 | \*\*\*\* | \*\*\*\* | 0.2007 | \*\*\*\* | \*\*\*\* | -0.0236 | \*\*\*\* | \*\*\*\* | -0.0005 | ns | ns | -0.0598 | \*\*\*\* | \*\*\*\* | -0.0166 | \*\*\*\* | \*\*\*\* | -0.0553 | \*\*\*\* | \*\*\*\* |
| Y | -0.0028 | ns | ns | -0.0841 | \*\*\*\* | \*\*\*\* | 0.0171 | \*\*\*\* | \*\*\*\* | -0.0184 | \*\*\*\* | \*\*\*\* | 0.0825 | \*\*\*\* | \*\*\*\* | 0.0118 | \*\*\*\* | \*\*\*\* | -0.0326 | \*\*\*\* | \*\*\*\* | 0.0343 | \*\*\*\* | \*\*\*\* | 0.0652 | \*\*\*\* | \*\*\*\* |
Table S5. Correlations between CDR3 length and amino acid usage across VHH regions in Bactrian.

## Slide 7
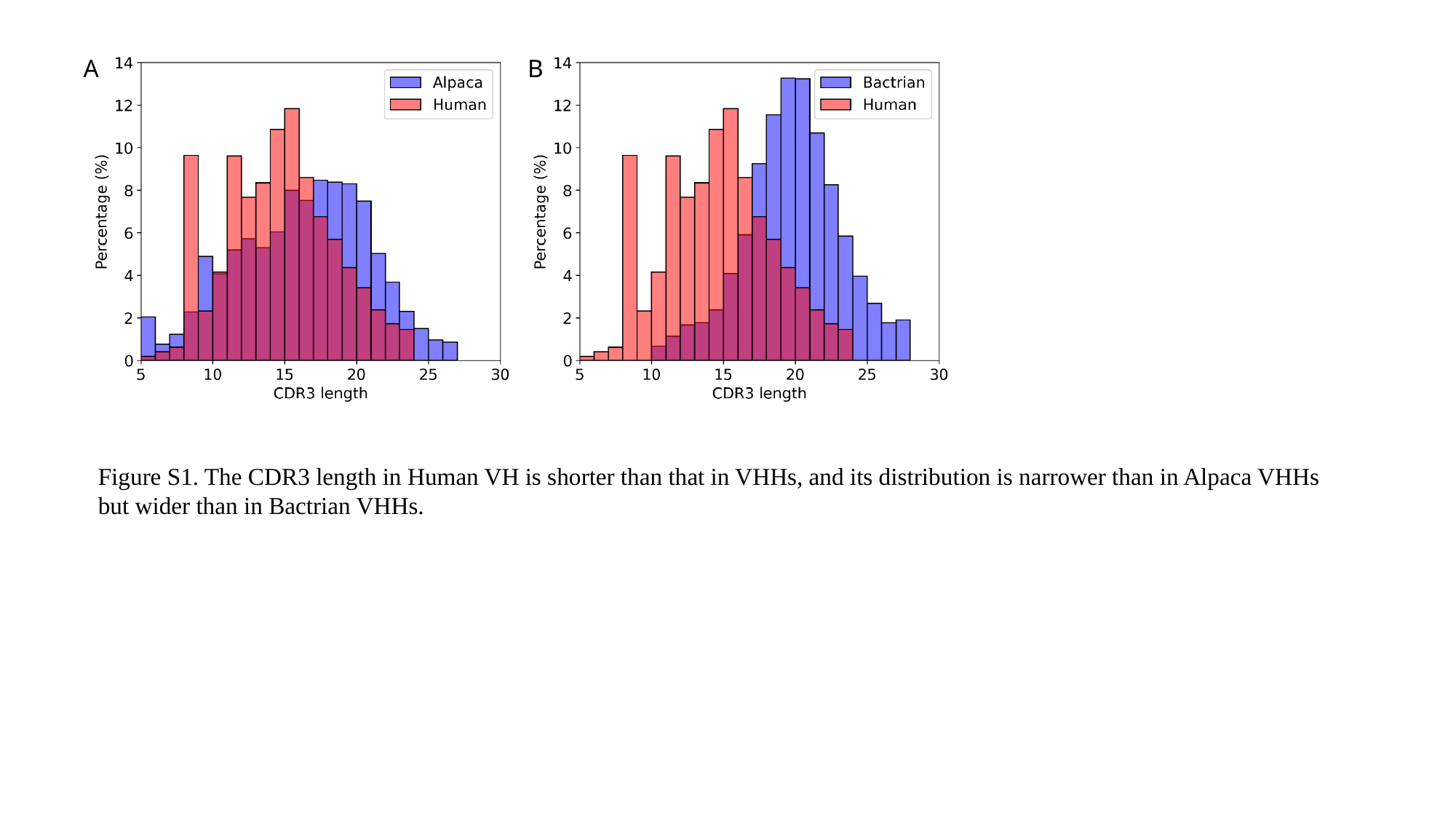

A
B
Figure S1. The CDR3 length in Human VH is shorter than that in VHHs, and its distribution is narrower than in Alpaca VHHs but wider than in Bactrian VHHs.

## Slide 8
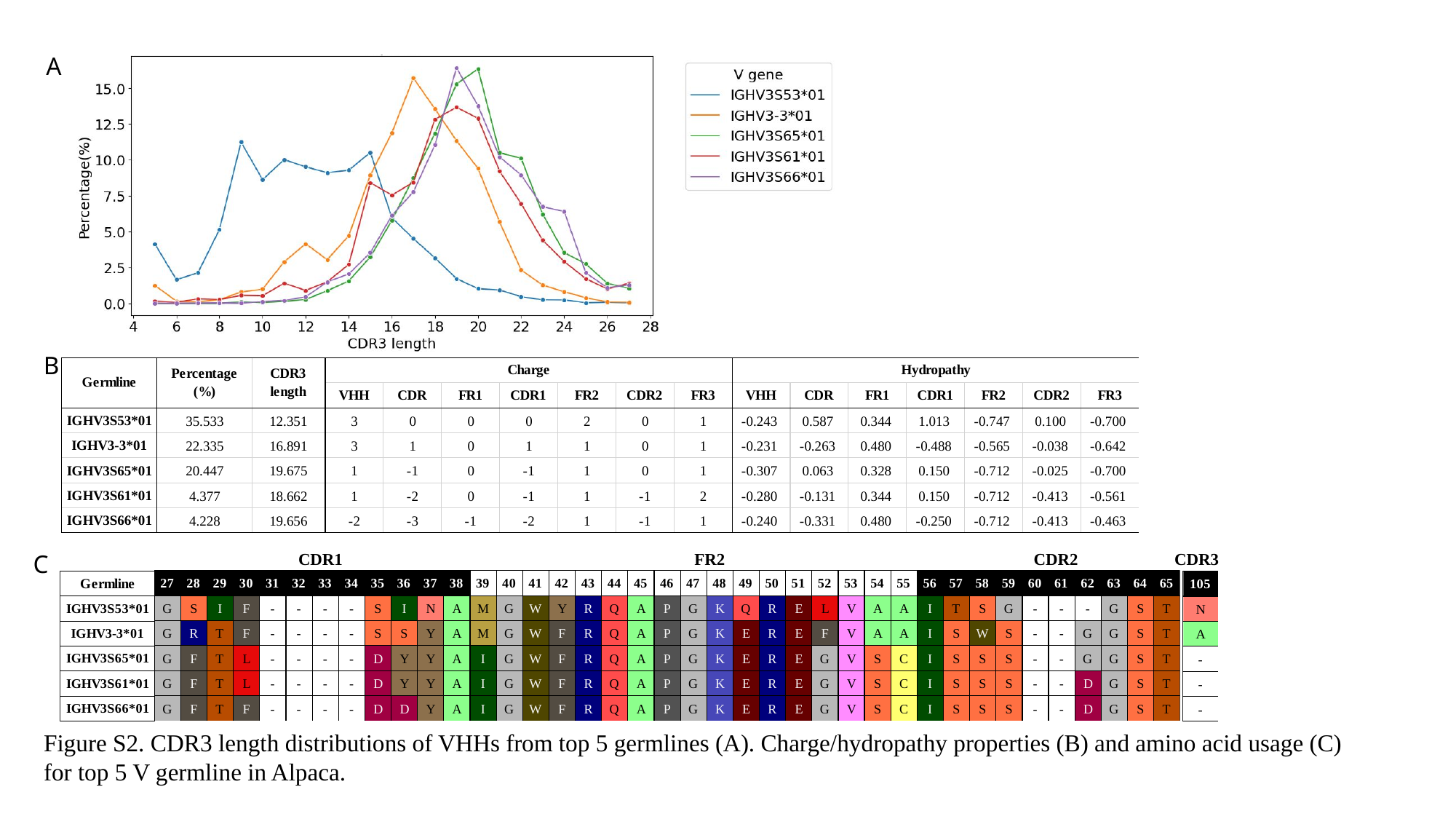

A
B
FR2
CDR2
CDR3
CDR1
C
Figure S2. CDR3 length distributions of VHHs from top 5 germlines (A). Charge/hydropathy properties (B) and amino acid usage (C) for top 5 V germline in Alpaca.

## Slide 9
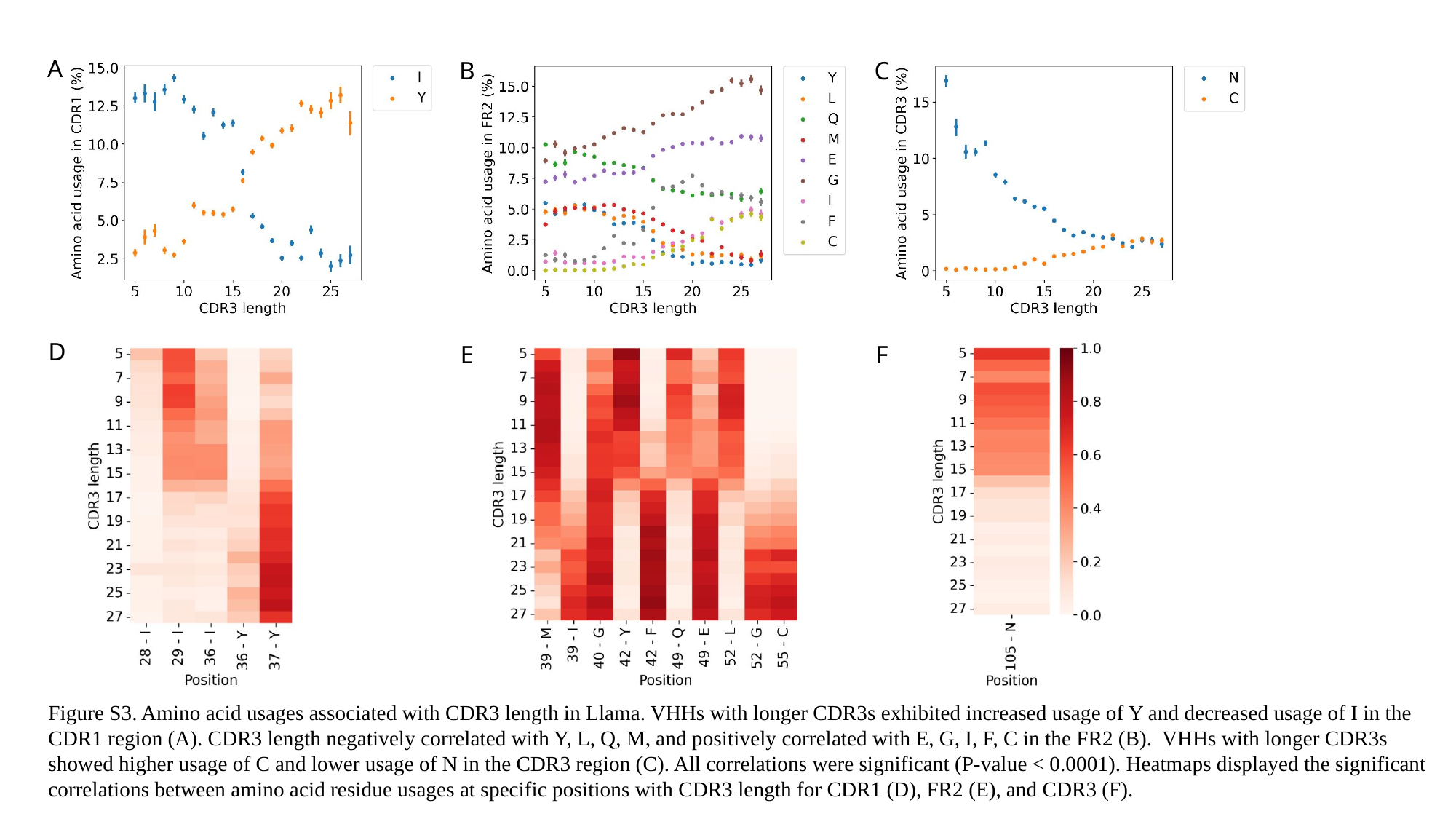

A
B
C
D
E
F
Figure S3. Amino acid usages associated with CDR3 length in Llama. VHHs with longer CDR3s exhibited increased usage of Y and decreased usage of I in the CDR1 region (A). CDR3 length negatively correlated with Y, L, Q, M, and positively correlated with E, G, I, F, C in the FR2 (B). VHHs with longer CDR3s showed higher usage of C and lower usage of N in the CDR3 region (C). All correlations were significant (P-value < 0.0001). Heatmaps displayed the significant correlations between amino acid residue usages at specific positions with CDR3 length for CDR1 (D), FR2 (E), and CDR3 (F).

## Slide 10
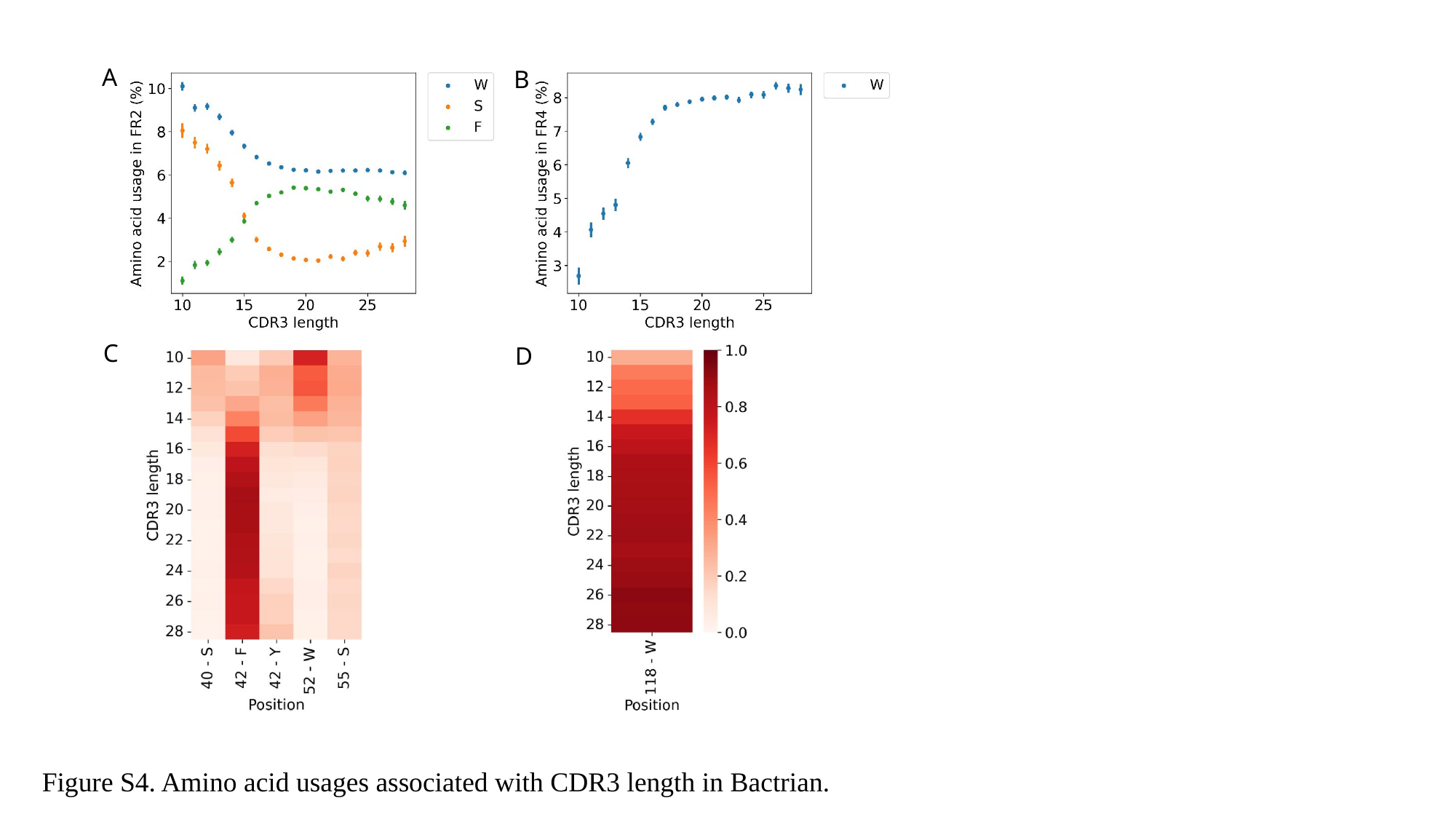

A
B
C
D
Figure S4. Amino acid usages associated with CDR3 length in Bactrian.

## Slide 11
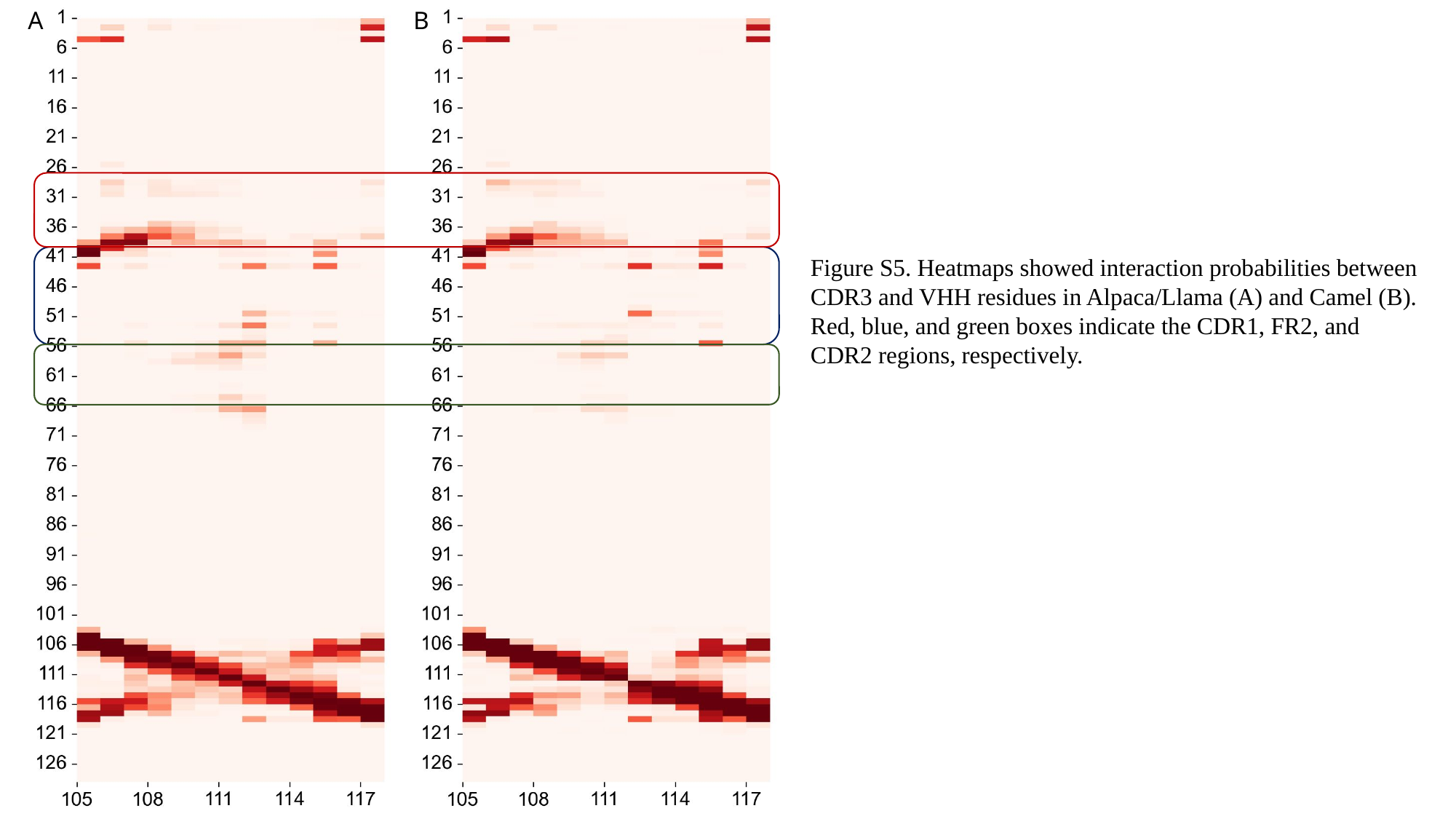

A
B
Figure S5. Heatmaps showed interaction probabilities between CDR3 and VHH residues in Alpaca/Llama (A) and Camel (B). Red, blue, and green boxes indicate the CDR1, FR2, and CDR2 regions, respectively.

## Slide 12
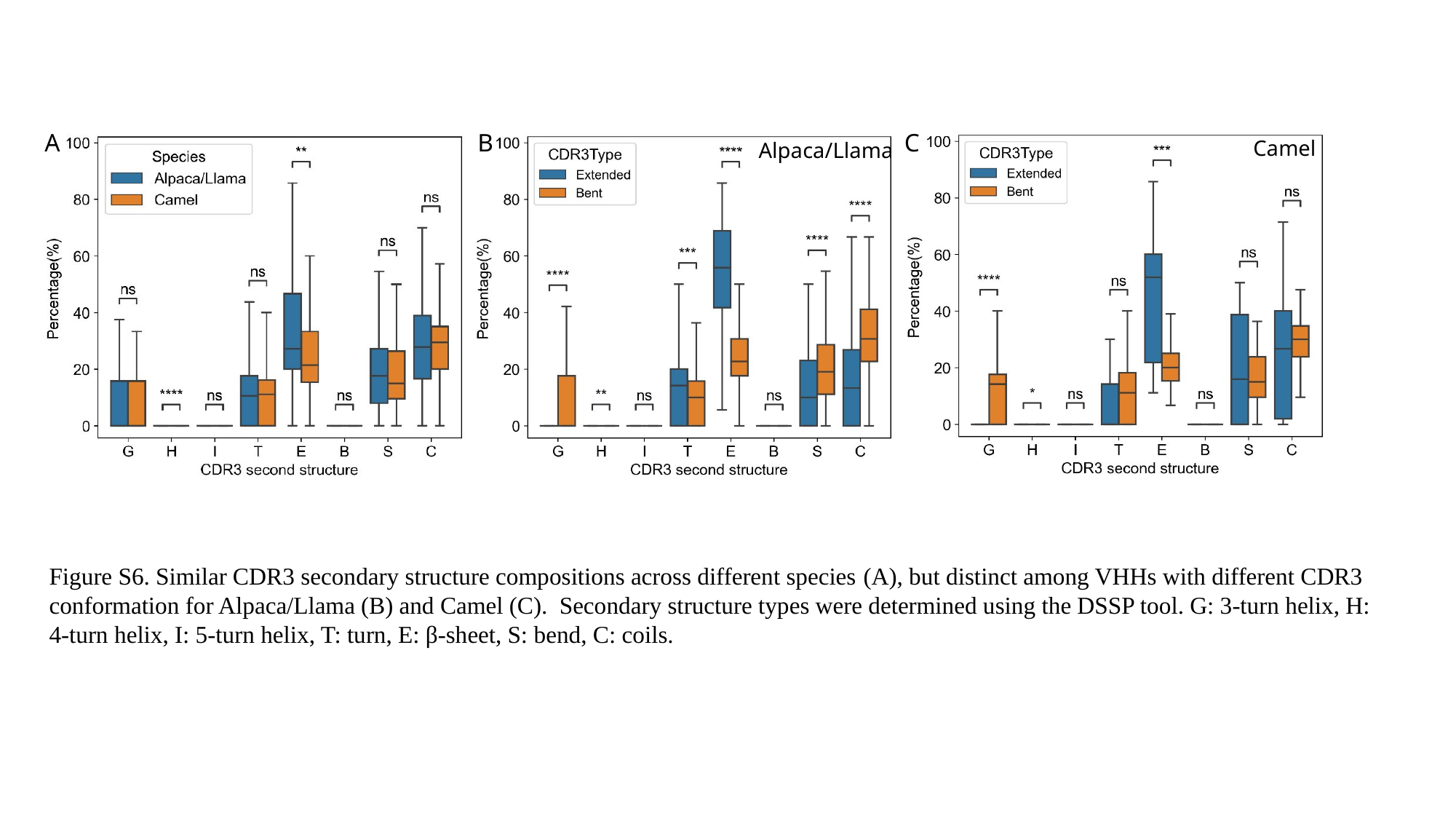

A
B
C
Camel
Alpaca/Llama
Figure S6. Similar CDR3 secondary structure compositions across different species (A), but distinct among VHHs with different CDR3 conformation for Alpaca/Llama (B) and Camel (C). Secondary structure types were determined using the DSSP tool. G: 3-turn helix, H: 4-turn helix, I: 5-turn helix, T: turn, E: β-sheet, S: bend, C: coils.

## Slide 13
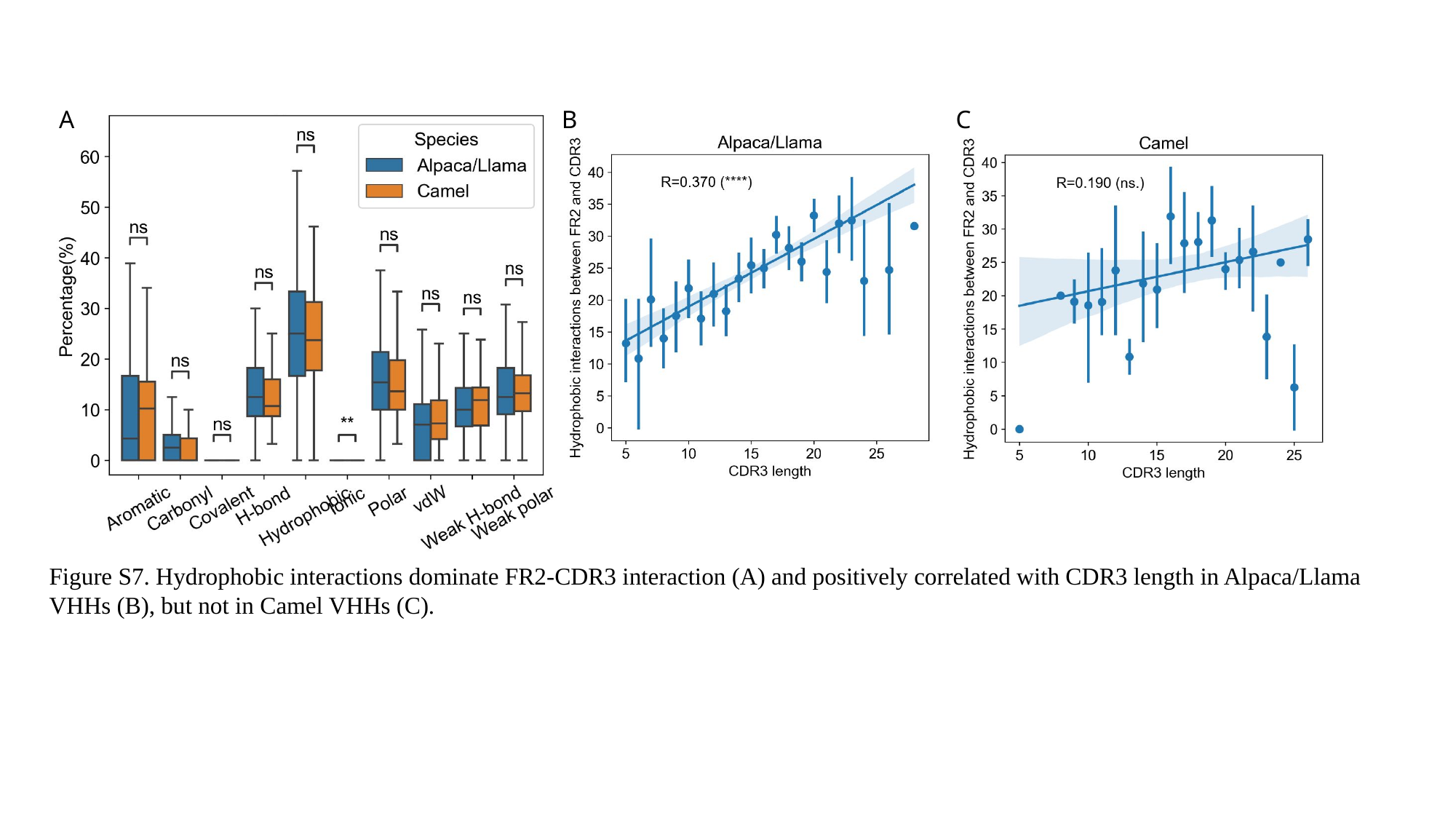

A
B
C
Figure S7. Hydrophobic interactions dominate FR2-CDR3 interaction (A) and positively correlated with CDR3 length in Alpaca/Llama VHHs (B), but not in Camel VHHs (C).

## Slide 14
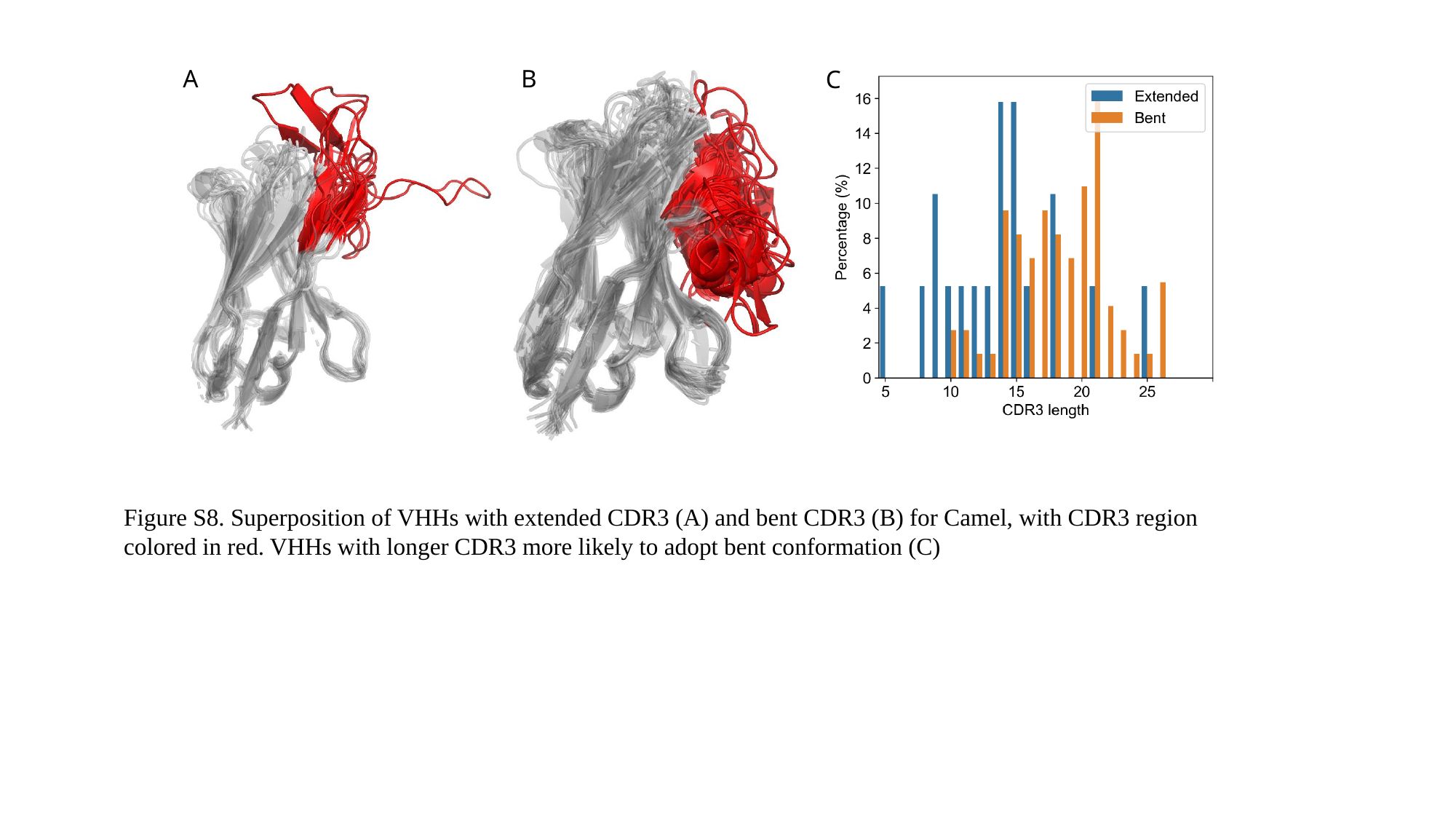

A
B
C
Figure S8. Superposition of VHHs with extended CDR3 (A) and bent CDR3 (B) for Camel, with CDR3 region colored in red. VHHs with longer CDR3 more likely to adopt bent conformation (C)

## Slide 15
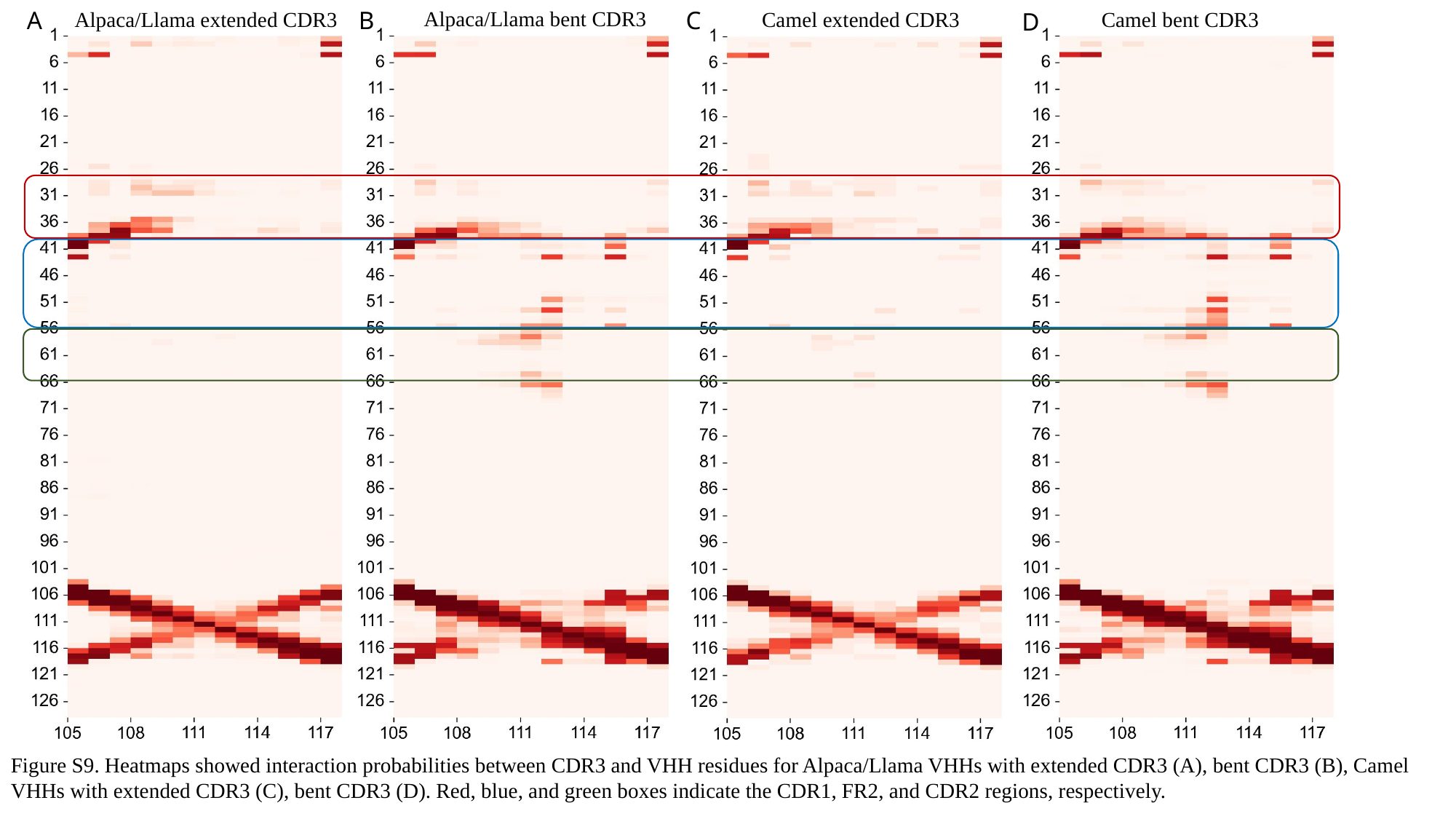

B
Alpaca/Llama bent CDR3
A
C
Camel extended CDR3
Camel bent CDR3
Alpaca/Llama extended CDR3
D
Figure S9. Heatmaps showed interaction probabilities between CDR3 and VHH residues for Alpaca/Llama VHHs with extended CDR3 (A), bent CDR3 (B), Camel VHHs with extended CDR3 (C), bent CDR3 (D). Red, blue, and green boxes indicate the CDR1, FR2, and CDR2 regions, respectively.

## Slide 16
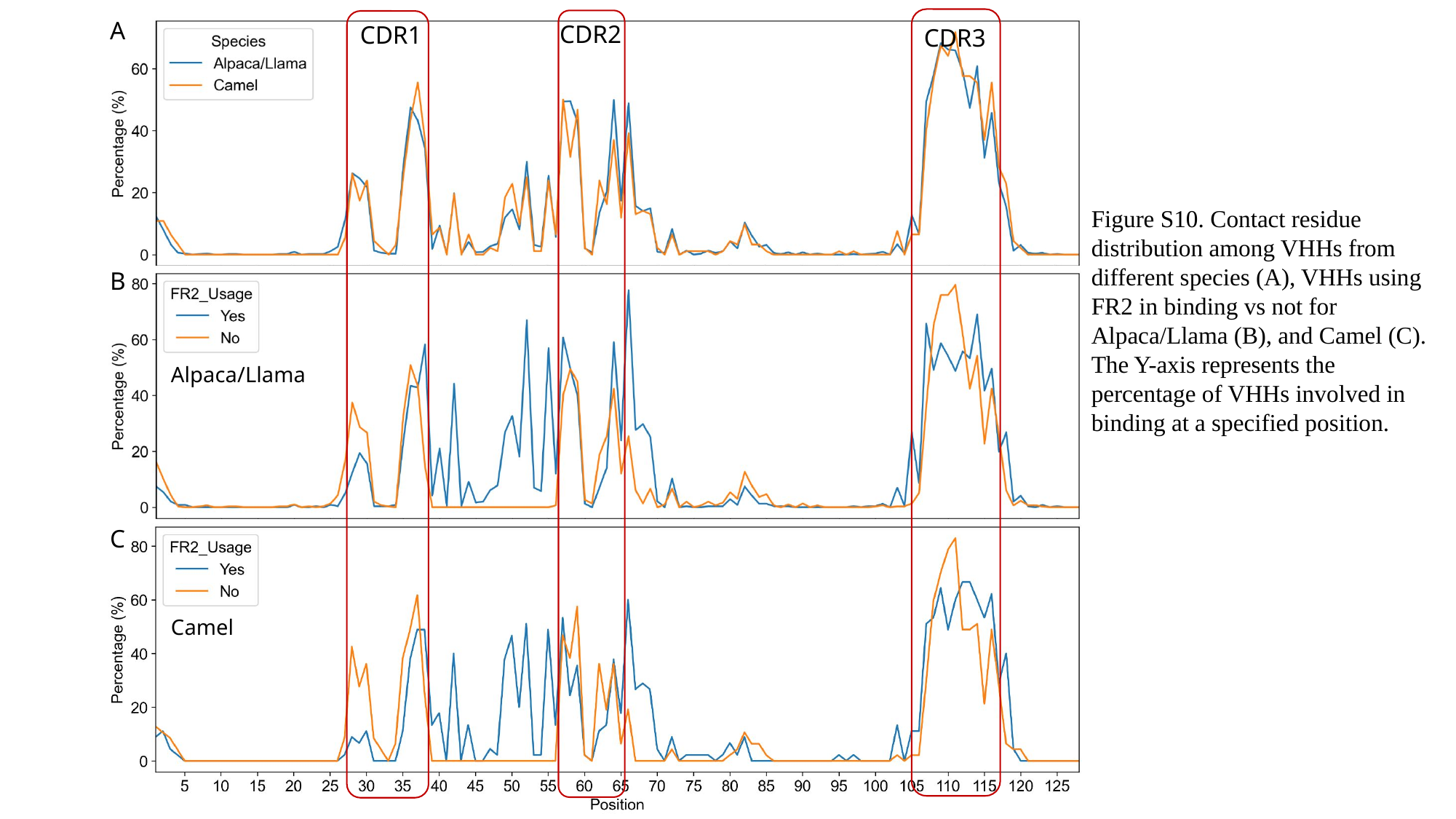

A
CDR2
CDR1
CDR3
Figure S10. Contact residue distribution among VHHs from different species (A), VHHs using FR2 in binding vs not for Alpaca/Llama (B), and Camel (C). The Y-axis represents the percentage of VHHs involved in binding at a specified position.
B
Alpaca/Llama
C
Camel

## Slide 17
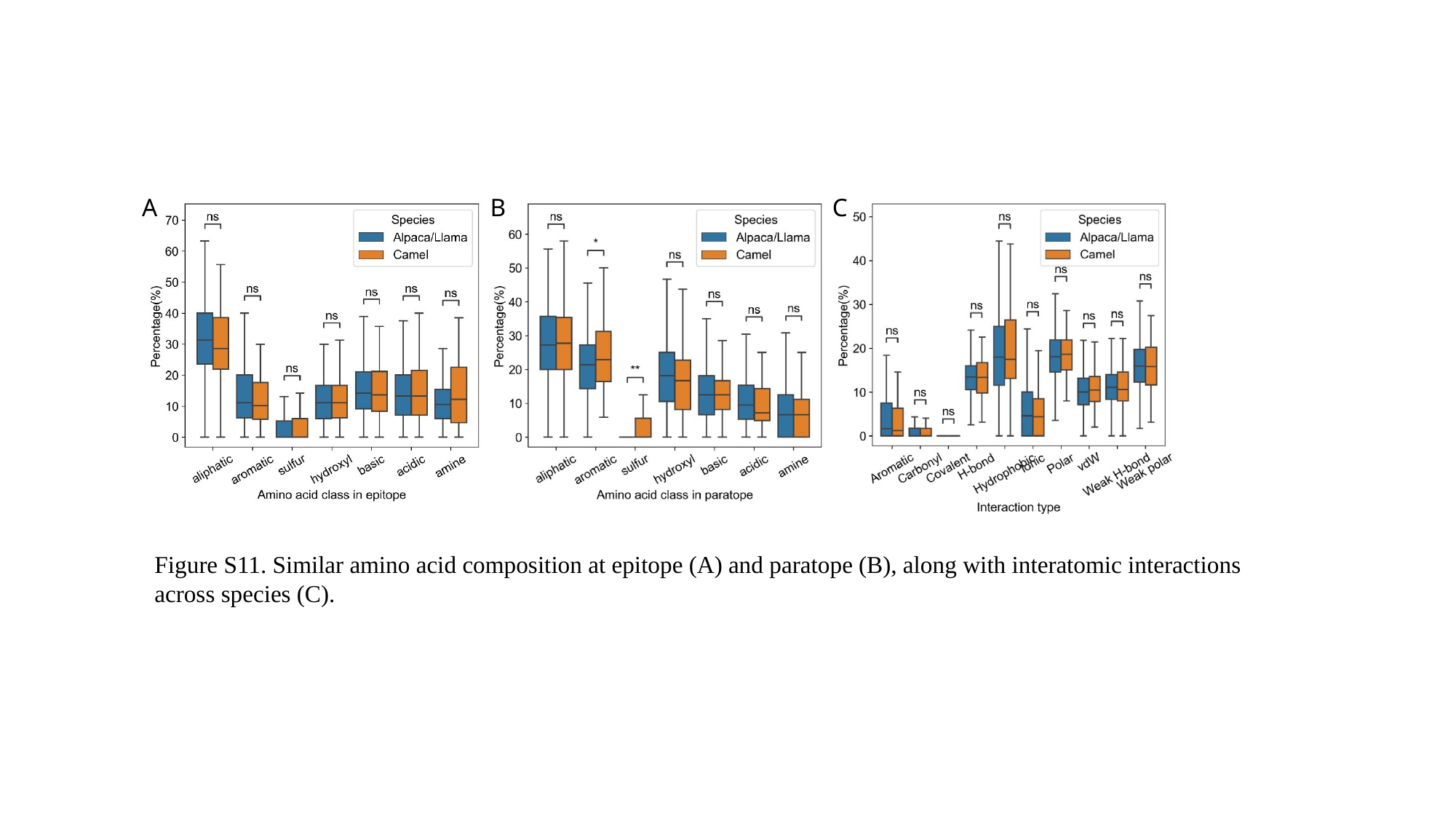

A
B
C
Figure S11. Similar amino acid composition at epitope (A) and paratope (B), along with interatomic interactions across species (C).

## Slide 18
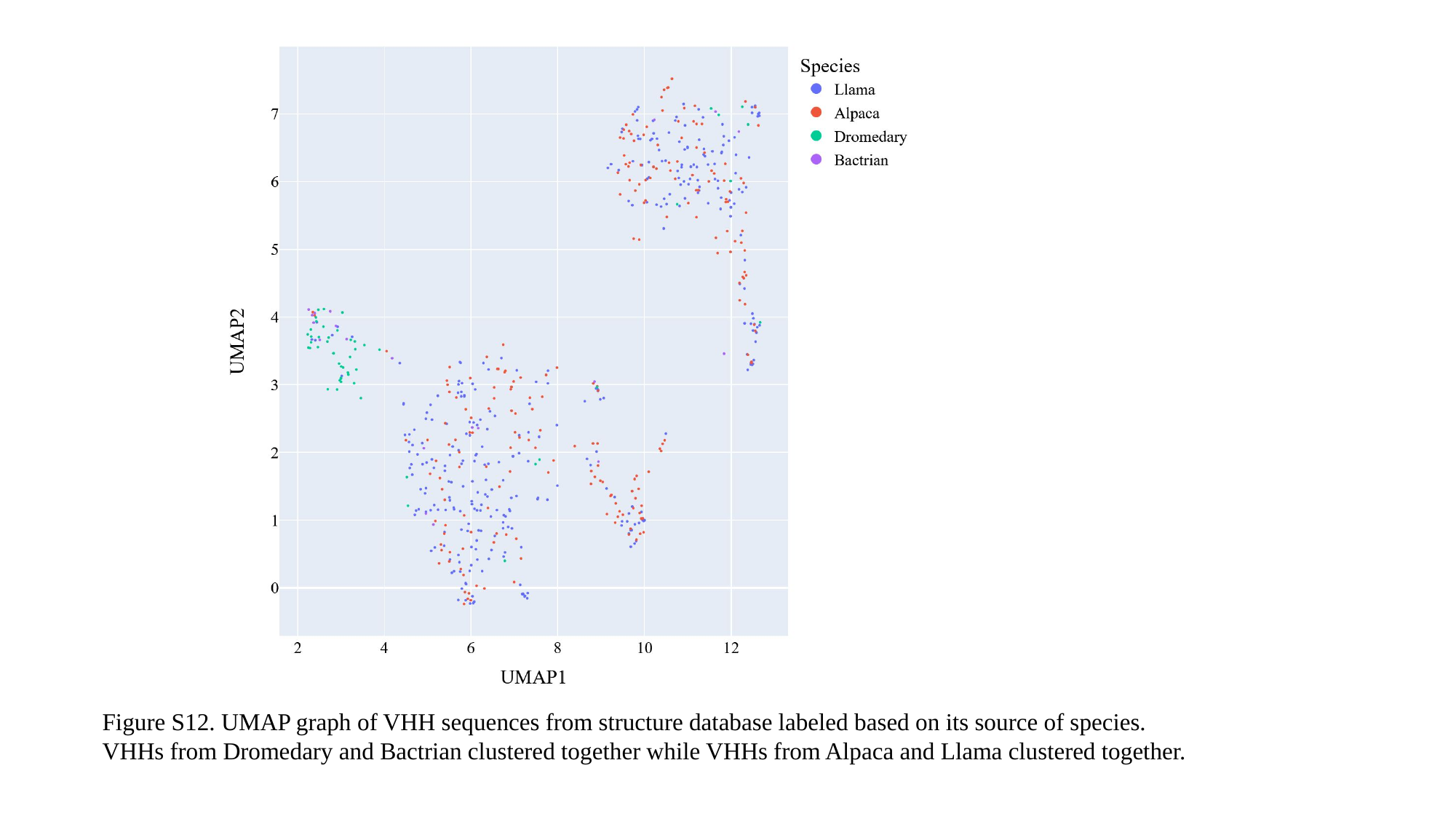

Figure S12. UMAP graph of VHH sequences from structure database labeled based on its source of species. VHHs from Dromedary and Bactrian clustered together while VHHs from Alpaca and Llama clustered together.

## Slide 19
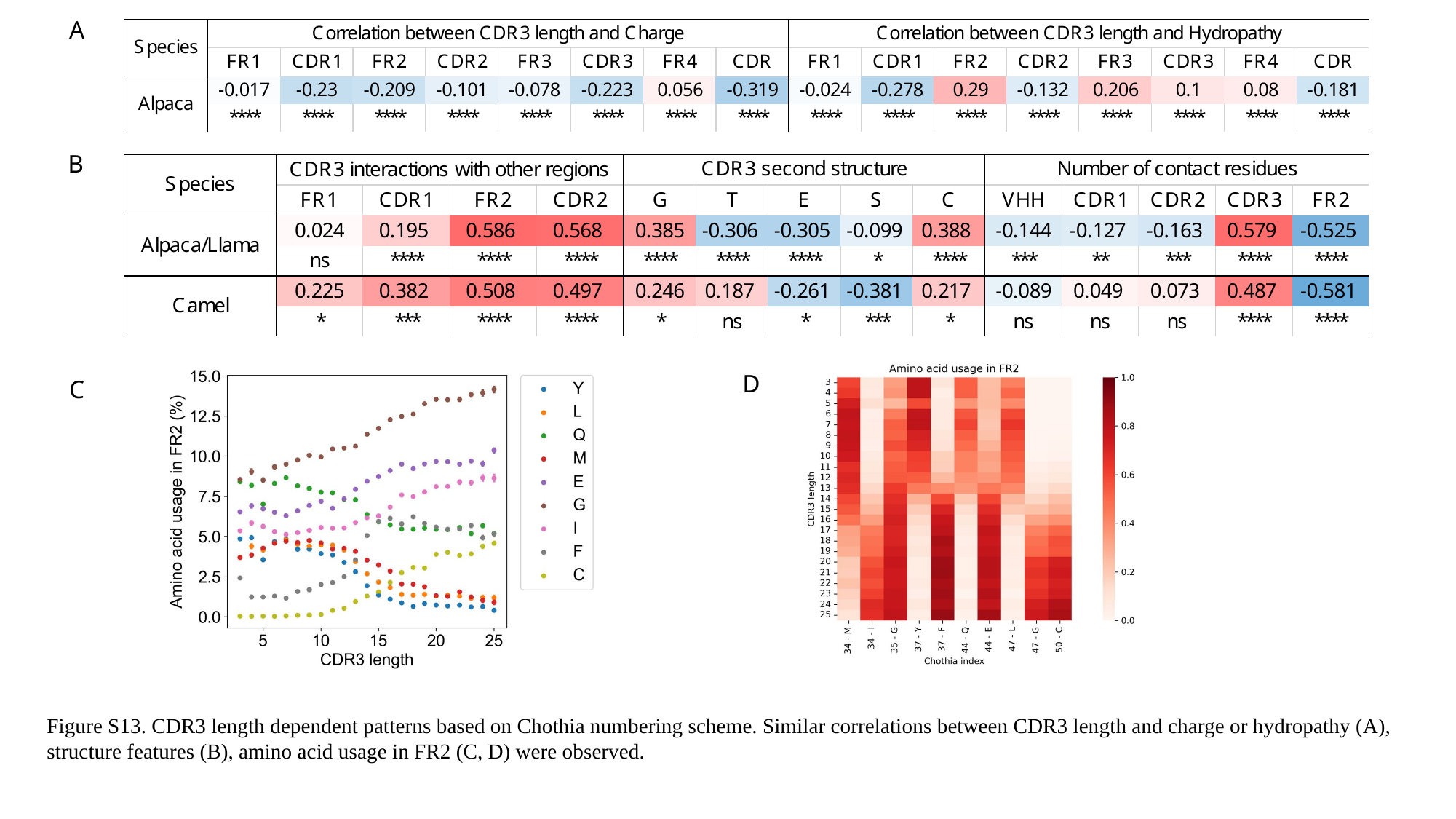

A
B
D
C
Figure S13. CDR3 length dependent patterns based on Chothia numbering scheme. Similar correlations between CDR3 length and charge or hydropathy (A), structure features (B), amino acid usage in FR2 (C, D) were observed.

## Slide 20
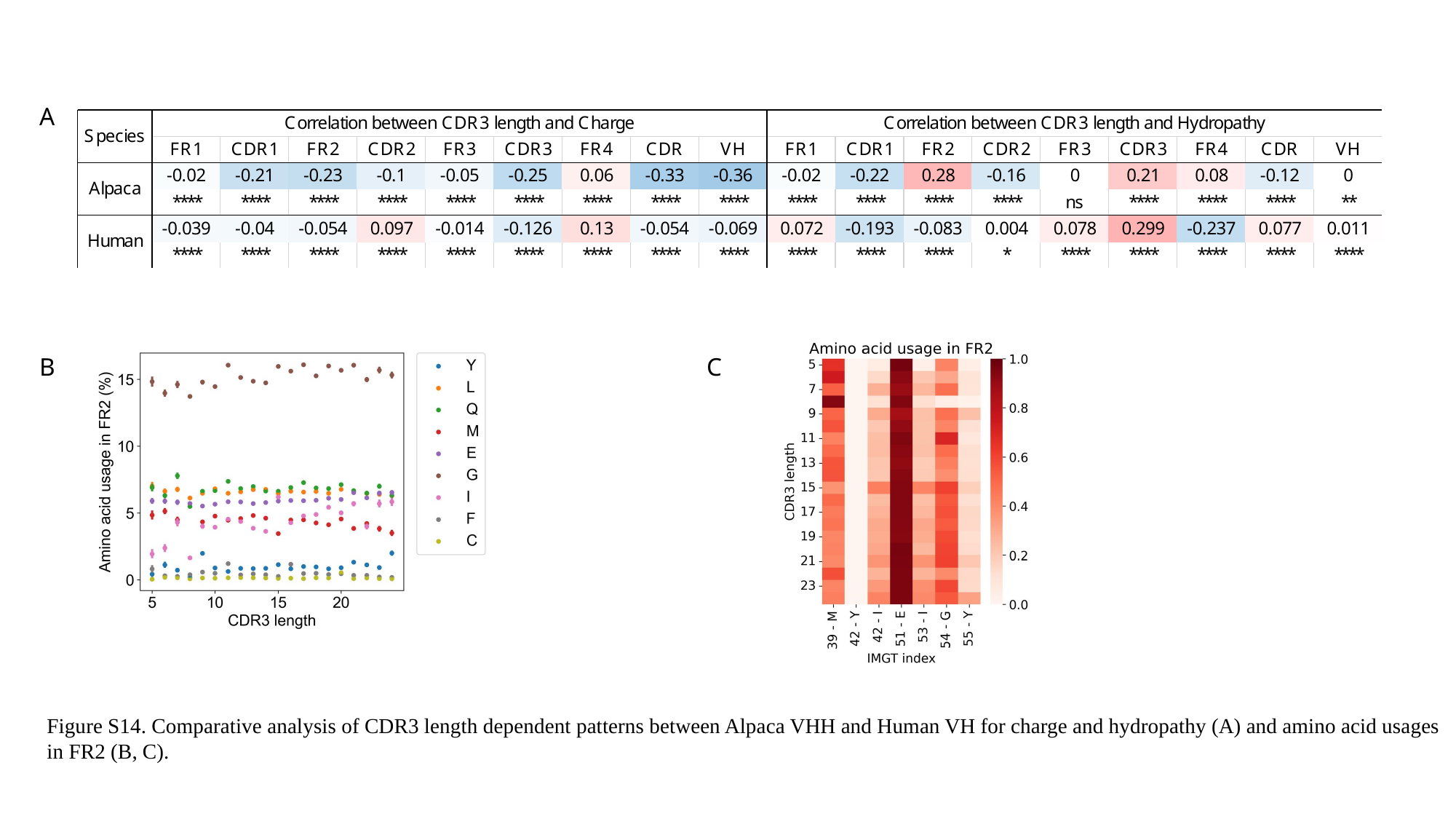

A
B
C
Figure S14. Comparative analysis of CDR3 length dependent patterns between Alpaca VHH and Human VH for charge and hydropathy (A) and amino acid usages in FR2 (B, C).
